# Multi–Cell Line Analysis of Lysosomal Proteomes Reveals Unique Features and Novel Lysosomal Proteins

**DOI:** 10.1016/j.mcpro.2023.100509

**Published:** 2023-02-14

**Authors:** Fatema Akter, Sara Bonini, Srigayatri Ponnaiyan, Bianca Kögler-Mohrbacher, Florian Bleibaum, Markus Damme, Bernhard Y. Renard, Dominic Winter

**Affiliations:** 1 Institute for Biochemistry and Molecular Biology, Medical Faculty, University of Bonn, Bonn, Germany; 2 Department of Pharmacology, Faculty of Veterinary Science, Bangladesh Agricultural University, Mymensingh, Bangladesh; 3 Bioinformatics Unit (MF1), Robert Koch Institute, Berlin, Germany; 4 Institute for Biochemistry, University of Kiel, Kiel, Germany

**Keywords:** lysosomes, proteomics, mass spectrometry, superparamagnetic iron oxide nanoparticles (SPIONs), posterior probability analysis, ACN, acetonitrile, DDA, data-dependent acquisition, DIA, data-independent acquisition, DMEM, Dulbecco’s modified Eagle’s medium, ER, endoplasmic reticulum, FA, formic acid, FCS, fetal calf serum, FDR, false discovery rate, GO, gene ontology, iBAQ, intensity-based absolute quantification, MS/MS, tandem mass spectrometry, PNS, postnuclear supernatant, RT, room temperature, SILAC, stable isotope labeling by amino acids in cell culture, SPION, superparamagnetic iron oxide nanoparticle

## Abstract

Lysosomes, the main degradative organelles of mammalian cells, play a key role in the regulation of metabolism. It is becoming more and more apparent that they are highly active, diverse, and involved in a large variety of processes. The essential role of lysosomes is exemplified by the detrimental consequences of their malfunction, which can result in lysosomal storage disorders, neurodegenerative diseases, and cancer. Using lysosome enrichment and mass spectrometry, we investigated the lysosomal proteomes of HEK293, HeLa, HuH-7, SH-SY5Y, MEF, and NIH3T3 cells. We provide evidence on a large scale for cell type–specific differences of lysosomes, showing that levels of distinct lysosomal proteins are highly variable within one cell type, while expression of others is highly conserved across several cell lines. Using differentially stable isotope-labeled cells and bimodal distribution analysis, we furthermore identify a high confidence population of lysosomal proteins for each cell line. Multi–cell line correlation of these data reveals potential novel lysosomal proteins, and we confirm lysosomal localization for six candidates. All data are available *via* ProteomeXchange with identifier PXD020600.

## Highlights

• Analysis of lysosome-enriched fractions/whole cell lysates from six cell lines.
• High dynamic range of lysosomal proteome.
• SILAC labeled background cells enable identification of enriched proteins.
• Definition of high confidence potential novel lysosomal proteins.
• Validation of six lysosomal candidate proteins by immunostaining.

## Graphical Abstract

**Figure:**
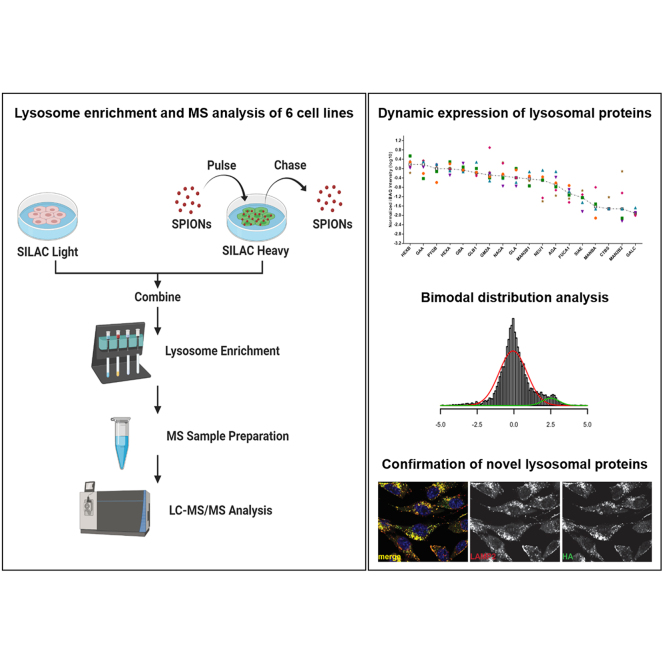

## In Brief

We present a comprehensive analysis of lysosome-enriched fractions from six commonly used mammalian cell lines. We show that lysosomal proteins are highly dynamic with respect to their expression and/or localization at lysosomes and provide a novel strategy for the identification of specifically enriched proteins. Based on the correlation of data from all cell lines, we identify high confidence novel lysosomal proteins and confirm six selected candidates.

Lysosomes, the central lytic organelles of the cell, are responsible for the degradation of a large variety of cellular compounds and the recycling of their building blocks, fulfilling a pivotal function for cellular homeostasis. Organelles and macromolecules, which are delivered to lysosomes through endosomes, phagosomes, and different forms of autophagy, are hydrolyzed by ∼60 enzymes residing in the lysosomal lumen (1). Defects in degradation, export, or trafficking of these enzymes frequently result in lysosomal dysfunction, causing the so-called lysosomal storage disorders, a group of rare inherited diseases with detrimental consequences for the affected patients (2). Furthermore, in the recent years, lysosomes have been shown to also play a role in more common diseases like neurodegenerative disorders (3) or cancer (4).

In addition to lysosomal hydrolases, >100 lysosomal integral and membrane-associated proteins have been confirmed to date. They are involved in a large range of processes including the transport of molecules across the lysosomal membrane, the fusion with vesicles, nutrient sensing, lysosomal positioning, and interaction of lysosomes with other organelles, *e.g.*, for the exchange of metabolites (5, 6). Aside from the mammalian target of rapamycin complex 1 (*mTORC1*), whose members and functions have been investigated in a variety of studies (reviewed in (7, 8)), the function of many lysosomal membrane proteins, and the composition of lysosome-associated complexes, remains insufficiently characterized. Expression of a large number of lysosomal proteins is regulated by members of the microphthalmia (MiT/TFE) family of transcription factors (9). The most prominent members of this group are transcription factors EB and E3 (TFEB, TFE3), which are frequently referred to as master regulators of lysosomal function and autophagy. Their transcriptional activity, and therefore the expression of lysosomal proteins, is regulated by direct phosphorylation through several kinases, which are important players of cellular signaling, such as mTOR, GSK3B, Erk1/2, PKC, or AKT (10). These kinases can induce lysosomal protein expression in response to different types of cellular stress such as amino acid/lipid starvation, protein aggregation, or exposure to microbe-associated molecular patterns (in the case of macrophages) (11, 12).

It is by now well established that lysosomes are involved in the degradation of cellular macromolecules as well as a plethora of other fundamental cellular processes. These include, for example, signaling, energy metabolism, protein secretion, antigen presentation, and plasma membrane repair (13), indicating that the number of proteins located in or at the lysosomal membrane may be well beyond those that are so far experimentally validated. In addition, it is known for a long time that lysosomes can differ between individual cell types (14), and, more recently, it is emerging that lysosomes can be subclassified into individual populations, differing, *e.g.*, in their pH value or their mobility (15, 16). Especially for cells of the immune system, such as macrophages or dendritic cells, it was shown that lysosomal morphology can change in response to cellular needs and, *e.g.*, exposure to pathogens, and that lysosomal function varies depending on the cell type (reviewed in (17)). Given the various roles lysosomes play in these cells, such as digestion of microbes, presentation of antigens, or turnover of endogenous material, it is certainly conceivable that they present unique properties depending on their function, and therefore a unique repertoire of proteins.

In order to be able to assess such differences on a global scale, and to identify novel lysosomal proteins, unbiased large-scale approaches play a pivotal role. The method that is currently used most frequently is mass spectrometry–based proteomics, as it allows to simultaneously identify and quantify large numbers of proteins in a given sample. A variety of studies have been performed, which investigated lysosomes and lysosomal proteins in HEK293 (18, 19, 20), HeLa (21, 22), and MEF (23) cells as well as lysosomes derived from tissue (24, 25, 26), and primary cells (27, 28). While the majority of these studies investigated a biological question, such as the alteration of the lysosomal proteome under pathological conditions (27, 28), some also focused on the identification of novel lysosomal proteins or the improvement/comparison of techniques for the enrichment of lysosomes, which is a crucial step for the untargeted proteomic analysis of lysosomal proteins due to their low abundance (18, 20, 23, 24, 29).

A common feature of datasets generated from lysosome-enriched fractions with sensitive state-of-the-art mass spectrometry approaches, is that a high number of identified proteins are apparently not lysosomal. In a recent study from our group, for example, >7000 proteins were identified in samples enriched for lysosomes using superparamagnetic iron oxide nanoparticles (SPIONs) (23), which is >60% of the proteins currently estimated to be expressed on average in *in vitro* cultivated cells (30), making it highly unlikely that all of them are related in one way or the other to lysosomes. Also for other approaches, as, *e.g.*, the enrichment of lysosomes by sucrose density gradient centrifugation or immunoprecipitation, high numbers of contaminating proteins (based on their gene ontology [GO] classification) have been identified (18), which most likely bind unspecifically to the beads or columns utilized for the enrichment of lysosomes.

While it is common practice to assess the likelihood of localization to a specific organelle in differential centrifugation approaches by comparison with other fractions (24, 31), to our knowledge, with the exception of one study, which assessed the unspecific binding of nonlysosomal proteins to anti-HA beads by label-free quantification (20), no attempts have been made to discriminate between lysosome-specific and unspecific binding to the affinity columns/beads. This leaves the question largely unanswered which of the proteins detected in such experiments are truly lysosomal. Furthermore, the vast majority of studies dealing with the proteomic analysis of lysosomes that were enriched from cell lines investigated only a single type of cells. Owing to the high variability achieved among individual lysosome enrichment methods and strategies for sample analysis by mass spectrometry–based proteomics, even for the same cell line (18), a comparison of lysosomal proteomes between different cell lines based on published data is therefore not straightforward.

In the current study, we investigated the lysosomal proteome of six widely used cell lines using lysosome enrichment by SPIONs and mass spectrometry–based proteomics (19). For three of them, we present the first draft of their lysosomal proteome. By comparison of expression levels for lysosomal proteins of the individual cell lines, we identify cell type–specific expression patterns, revealing lysosomal heterogeneity on a global scale. For the identification of truly lysosomal proteins, we developed a novel approach for the definition of background proteins, based on the combination of SPIONs, stable isotope labeling by amino acids in cell culture (SILAC), and a bimodal distribution model for data analysis. Finally, we show that the reproducibility of protein identification across cell lines correlates with the likelihood of lysosomal localization, proposing potential novel lysosomal proteins and confirming lysosomal localization for selected candidates.

## Experimental Procedures

### Cell Culture and Stable Isotope Labeling by Amino Acids in Cell Culture

HEK293 (Catalog no. CRL-1573, ATCC), HeLa (Catalog no. CCL-2, ATCC), HuH-7 (Catalog no. JCRB0403, JCRB Cell Bank), SH-SY5Y (Catalog no. CRL-2266, ATCC), MEF (Catalog no. CRL-2991, ATCC), and NIH3T3 (Catalog no. CRL-1658, ATCC) cells were cultured in SILAC Dulbecco’s modified Eagle’s medium (DMEM) supplemented with 10% dialyzed fetal calf serum (FCS), 100 IU/ml penicillin, 100 μg/ml streptomycin, 181.2 mg/ml light or ^13^C_6_^15^N_2_ lysine (Catalog no. CNLM-291-H-1, Cambridge Isotope Laboratories, Inc), and 87.8 mg/ml light or ^13^C_6_^15^N_4_ arginine (Catalog no. CNLM-539-H-1, Cambridge Isotope Laboratories, Inc) at 37 °C, 100% humidity, and 5% CO_2_. Each cell line was passaged at least six times to ensure complete SILAC labeling.

### Enrichment of Lysosomes from Different Cell Lines

For lysosome enrichment of MEF, NIH3T3, HeLa, and HuH-7 cells, 3 × 10^6^ cells were seeded per 10-cm plate and cultured in DMEM with 2.5% FCS for 72 h. Subsequently, the medium was changed to DMEM with 10% FCS including 10% of SPIONs solution (Catalog no. BKS25, DexoMAG40, Liquids Research Ltd) for either light or heavy SILAC labeled cells (two replicates each), followed by incubation for 24 h. For HEK293 cells, plates were coated with 100 μg/ml poly-L-lysine (Catalog no. P1524, Sigma-Aldrich). For HEK293 and SH-SY5Y cells, 6 × 10^6^ cells were seeded directly in DMEM with 10% FCS and 10% SPIONs followed by incubation for 24 h. Subsequently, for all cell lines, cells were washed twice with 1× PBS, fresh medium was added, and the cells were incubated for 36 h. Cells were washed three times with ice-cold 1× PBS, scraped in 2 ml isolation buffer (250 mM sucrose, 10 mM Hepes/NaOH pH 7.4, 15 mM KCl, 1 mM CaCl_2_, 1 mM MgCl_2_, 1.5 mM MgAc, 1 mM dithiothreitol [DTT], and 1× cOmplete EDTA-free protease inhibitor cocktail [Catalog no. 11873580001, Roche Diagnostics GmbH] per plate, and lysed using a 15-ml Dounce homogenizer. Nuclei and intact cells were pelleted by centrifugation at 4 °C, 500*g* for 10 min, and the postnuclear supernatant (PNS) was transferred to a new tube. The pellet was resuspended in 2 ml isolation buffer, the procedure was repeated, and the PNS fractions were combined. The pooled PNS fractions of the individual cell lines were passed by gravity flow through LS columns (Catalog no. 130-042-401, Miltenyi Biotech) placed in a MidiMACS Separator (Catalog no. 130–042–302, Miltenyi Biotech), and washed with 5 ml isolation buffer. Lysosomes were eluted from the columns twice with 1 ml isolation buffer each using a plunger. Lysosomal integrity and isolation efficiency were assessed using the β-hexosaminidase assay with/without the addition of 0.8% Triton X-100 (v/v, final concentration) (19). To calculate the relative recovery of lysosomes in the eluate fractions, only the contribution of cell lines receiving SPIONs was considered. The protein concentration was determined using the DC protein assay (Catalog no. 5000116, Bio-Rad).

### Sample Preparation for Mass Spectrometry

For each sample, 100 μg of protein was precipitated by ice-cold chloroform/methanol (2:1, v/v) as described (32). Protein pellets were resuspended in 8 M urea, 0.1 M TEAB (33), and incubated at room temperature (RT) for 45 min, 800 rpm followed by reduction with 5 mM dithiothreitol (DTT) at 56 °C, 800 rpm for 25 min, and alkylation with 20 mM acrylamide at RT for 30 min (34). The reaction was quenched by addition of 5 mM DTT, samples were diluted to 4 M urea with 0.1 M TEAB, rLys-C (Catalog no. V1671, Promega) added at an enzyme to protein ratio of 1 to 100 (w/w), and digestion was performed at 37 °C overnight. The following day, samples were diluted with 0.1 M TEAB to 1.6 M urea, trypsin (Catalog no. V5111, Promega) was added at an enzyme to protein ratio of 1 to 100 (w/w), and the samples were incubated at 37 °C for 10 h. Finally, the samples were acidified using acetic acid (0.1% final concentration) and 10 μg of peptides was desalted by STAGE tips as described (35). Eluted peptides were dried using a vacuum centrifuge and resuspended in 5% acetonitrile (ACN)/5% formic acid (FA).

### Liquid Chromatography–Tandem Mass Spectrometry Analysis

Mass spectrometry analyses were performed using a Dionex Ultimate 3000 system coupled to an Orbitrap Fusion Lumos mass spectrometer (both Thermo Scientific). Columns were manufactured in-house as follows: 50-cm spray tips were generated from 360 μm outer diameter/100 μm inner diameter fused silica capillaries using a P-2000 laser puller (Sutter Instruments) and packed with 1.9 μm or 3 μm Reprosil AQ C_18_ particles (Catalog no. r119.aq and r13.aq, Dr. Maisch) for data-dependent acquisition (DDA) or data-independent acquisition (DIA) analyses, respectively. Peptides were resuspended in 5% ACN/5% FA and loaded on the analytical column at a flow rate of 600 nl/min, 100% solvent A (0.1% FA in water). Peptide separation was performed at a flow rate of 300 nl/min with a 240-min linear gradient (DDA) or a 120-min gradient (DIA) from 5 to 35% solvent B (95% ACN/0.1% FA). Survey spectra were acquired in the Orbitrap mass analyzer with a mass range of *m/z* 375 to 1575 at a resolution of 60,000. For DDA analyses, tandem mass spectrometry (MS/MS) fragmentation was performed for charge states between 2 and 4 by higher-energy collisional dissociation (HCD) with 30% collision energy and data were acquired in the Orbitrap at a resolution of 30,000. The cycle time was set to 5 s and the precursor isolation width to 1.6 *m/z* using the quadrupole. For mass spectrometry (MS) and MS/MS scans, the automatic gain control was set to 4 × 10^5^ and 5 × 10^5^, respectively. For DIA analyses, survey spectra were acquired in the Orbitrap mass analyzer with a mass range of *m/z* 350 to 1200 at a resolution of 120,000 and a maximum injection time of 50 ms. MS/MS was performed by isolation of *m/z* 24.1 windows using the quadrupole and higher-energy collisional dissociation fragmentation with 30% collision energy. Data were acquired in the Orbitrap at a resolution of 30,000, with a maximum injection time of 54 ms, and a fixed mass range of m/z 200 to 2000. The cycle time was set to 3 s, and the automatic gain control was set to 5 × 10^5^ and 1 × 10^6^ for MS and MS/MS spectra, respectively.

### Mass Spectrometry Data Analysis

DDA Thermo ∗.raw files were analyzed with Proteome Discoverer 2.2 (Thermo Fisher Scientific) in combination with Mascot 2.6.1 (www.matrixscience.com). For database searching, UniProt (Swiss-Prot + TrEMBL) *Homo sapiens* (release 2019_05, 73,920 entries) and UniProt *Mus musculus* (release 2019_05, 54,425 entries) in combination with the cRAP database (ftp://ftp.thegpm.org/fasta/cRAP/crap.fasta) including common contaminants were used. The following parameters were defined: variable modifications: oxidation of methionine, acetylation of protein N termini; fixed modification: propionamide at cysteine; mass tolerance: 10 ppm for precursor ions, 50 mmu for fragment ions; enzyme: trypsin except proline was the next amino acid; missed cleavage sites: two. Data were filtered with a false discovery rate (FDR) of 1% at the peptide level using Percolator (36), and proteins were exported with an FDR of 1%. For quantification, SPIONs/control ratios were determined for each individual raw file and unique as well as razor peptides were used. iBAQ values were calculated using MaxQuant version 1.6.14.0 in combination with the UniProt (Swiss-Prot + TrEMBL) reference proteomes for *H. sapiens* (release 2020_04, 96,808 entries) and *M. musculus* (release 2020_04, 63,666 entries). The same search parameters as for Proteome Discoverer were applied with the exception of definition of heavy labeled arginine (Arg10) and lysine (Lys8) as fixed modification for cells receiving SPIONs in the heavy SILAC channel. DIA data were analyzed by Spectronaut (version 16.2.220903.53000). DDA and DIA raw files were used to generate hybrid spectral libraries using UniProt (Swiss-Prot) *H. sapiens* (release 2021_01, 20,394 entries) or UniProt (Swiss-Prot) *M. musculus* (release 2021_01, 17,056 entries) including common contaminants. For each peptide, the three to six most abundant b/y ions were selected for library generation, depending on their signal intensity. The same fixed/variable modifications were used as in Proteome Discoverer and MaxQuant analyses. Data were filtered with the following q value cutoffs: precursor = 0.01, protein (experiment) = 0.01, protein (run) = 0.05. Dynamic retention time alignment was performed based on the high-precision indexed retention time concept (37). Quantification data were extracted on the MS2 level, followed by global median normalization and run-wise missing value imputation.

### Bioinformatics Analysis

Only peptides identified with high confidence were exported from Proteome Discoverer, MaxQuant, and Spectronaut for further analysis using R 3.5.1 (2018-07-02) (38), Microsoft Excel 2016, GraphPad Prism 6.07 (GraphPad Software), and Instant clue (v.0.10.10.20210316) (39). Abundance ratios of SILAC-labeled proteins (SPIONs/control) were log2-transformed and median-normalized using R. To estimate the parameters of the probability distribution, an expectation-maximization algorithm (EM algorithm) (40) was applied using the normalmixEM function of the R mix tools package (41). Setting the starting values (mu = (0.5) and sigma = (1.1)), estimators for mixtures of two univariate normal distributions were calculated, and a posterior probability was assigned to each protein by inserting the estimated parameters into the formula:

$$P\left(x\right)=\frac{P\left(x|mu_{2},sigma_{2}\right)}{P\left(x|mu_{1},sigma_{1}\right)+P\left(x|mu_{2},sigma_{2}\right)}$$

The derived probability describes the likelihood of the observation based on the calculated model. Extreme abundance ratio values (100 and 0.01) were handled separately, as they indicate missing values in one of the distributions and are arbitrarily set by Proteome Discoverer. Therefore, they were replaced with the maximal or minimal abundance ratios observed during data analysis and the posterior probabilities of these values were set to 1 or 0, respectively. For each dataset, the FDR was calculated applying the Benjamini–Hochberg (42) multiple testing procedure of R (https://cran.r-project.org/web/packages/BH) with a default significance level (alpha = 0.05). Principal component analysis was performed using the prcomp function of the R package stats, version 3.6.2, setting center = TRUE and scale = TRUE (38). Pearson correlation coefficients were calculated with the rcorr function from the R package Hmisc, version 4.4-0 (type = “pearson”) (https://CRAN.R-project.org/package=Hmisc). Heatmaps of abundance ratios and Pearson correlation were created with the heatmap.2 function from the R package gplots, version 3.0.3, and using hierarchical clustering (order = hclust) (http://cran.r-project.org/package=gplots). For UpSet plots, Proteome Discoverer data were joined using the full join function of the R package dplyr, version 0.7.4 (https://dplyr.tidyverse.org, https://github.com/tidyverse/dplyr). If a protein had been found in a cell line, the value was set to 1. Otherwise, if there was a missing value, the respective cell was set to 0. UpSet plots were generated with the upset function from the R package UpSetR, version 1.4.0, setting order.by = degree (43). For processing of iBAQ values, the median iBAQ intensity of V-ATPase subunits detected with ≥10 unique peptides was calculated and used to normalize the other proteins of the respective replicate. Subsequently, the median of the V-ATPase normalized values across individual replicates was calculated and utilized for further analyses. GO and functional enrichment analyses were performed using PANTHER (http://pantherdb.org/, database release date 2019-02-02) and g:Profiler (https://biit.cs.ut.ee/gprofiler/) (44, 45). Overrepresentation of subcellular localization was assessed with the GO cellular component complete function of PANTHER (*p* < 0.05, Benjamini–Hochberg, FDR corrected). For functional analysis of high confidence potential novel lysosomal proteins, the Gene Group Functional Profiling (g:GOSt) tool available in the g:Profiler web server (version from 2020-03–09) was used. Statistical enrichment analysis with g:GOSt was performed with GO molecular function, GO cellular component, GO biological process, and Reactome (*p* < 0.05, g:SCS threshold method).

### Experimental Design and Statistical Rationale

Samples for mass spectrometric analysis were generated for each cell line in four independent biological replicates. In order to address potential bias in the analysis of SILAC ratios, label switching was performed for the individual cell lines with two replicates receiving SPIONs in the light and heavy channels, respectively. In subsequent analyses, only proteins were considered with valid values in at least three biological replicates.

### RT-PCR and Molecular Cloning

RNA was isolated from MEFs or NIH3T3 cells using the Nucleospin RNA plus kit (Catalog no. 740984.5, Macherey-Nagel). Subsequently, cDNA was generated using the RevertAid First Strand Synthesis kit (Catalog no. K1621, Thermo Fisher Scientific) according to the manufacturer’s instructions utilizing random hexamer primers. Downstream RT-PCR amplification of lysosomal/endosomal genes was carried out with the respective set of primers (supplemental Table S14). PCR products were purified with the High Pure PCR Product Purification Kit (Catalog no. 11732676001, Sigma-Aldrich) and cloned into the pcDNA3.1 Hygro+ mammalian expression vector (Catalog no. V87020, Thermo Fisher Scientific). Successful cloning and sequence identity for the respective gene’s cDNA was confirmed using Sanger sequencing (Eurofins) for each generated plasmid. The Tspan3-myc expression vector was a kind gift from Paul Saftig, and the *Rab5-GFP* expression vector was a kind gift from Sergio Grinstein.

### Generation of NIH3T3 Cells Stably Expressing Lysosomal Candidate Proteins

NIH3T3 cells were seeded 24 h prior to transfection, and 4.5 μg of the respective plasmid DNA was transfected using TurboFect transfection reagent (Catalog no. R0534, Thermo Fisher Scientific). Cells were cultured for 24 h in DMEM with 10% FCS and 100 IU, ml penicillin/100 μg/ml streptomycin (Catalog no. 15140122, Thermo Fisher Scientific). Subsequently, the culture medium was replaced, fresh medium containing 500 μg/ml hygromycin B (Catalog no. H3274, Sigma-Aldrich) was added to the cells, and this step was repeated after 24 h. Before analysis, all transfected cells were cultured for a minimum of 4 weeks in DMEM containing 500 μg/ml hygromycin B.

### Immunostaining and Confocal Laser Microscopy

HeLa cells were cultured using cell culture medium as described above. Cells were seeded on glass coverslips 1 day prior to transfection. The transfection was carried out with 750 ng plasmid DNA using TurboFect and cells were cultured for 48 h before they were processed for immunostaining. Both transient (HeLa) and stable (NIH3T3) cells were washed three times with 1× PBS and fixated with either methanol or 4% paraformaldehyde in 1× PBS for 10 or 20 min, respectively. Subsequently, samples were rewashed three times with 1× PBS and incubated for 5 min in 1× PBS with 0.2% saponin, and for 15 min in 1× PBS with 0.02% glycine and 0.2% saponin. All samples were blocked for 1 h in 1× PBS with 0.2% saponin and 10% FCS. After blocking, cells were stained with primary antibodies against HA tag (Catalog no. 12158167001, 3F10, 1:100, Sigma-Aldrich), Myc tag (Catalog no. 2278, 71D10, 1:100, Cell signaling), and *LAMP2* (Catalog no. H4B4, DSHB, 1:100) overnight at 4 °C. The next day, all samples were repeatedly washed in 1× PBS with 0.2% saponin and incubated with the secondary antibodies donkey anti-rat Alexa Fluor 488 (Catalog no. A21208, Thermo Fisher Scientific), donkey anti-rabbit Alexa Fluor 488 (Catalog no. A21206, Thermo Fisher Scientific), and donkey anti-mouse Alexa Fluor 594 (Catalog no. A21203, Thermo Fisher Scientific) for 1 h at RT. After washing with 1× PBS, 0.2% saponin, coverslips were rinsed twice with H_2_O, and embedded in Mowiol-DABCO with DAPI (4-,6-diamidino-2-phenylindole) (Catalog no. 62247, Thermo Fisher Scientific). Transiently transfected cells were analyzed with an inverted confocal laser scanning microscope (Catalog no. FV1000, Olympus, Life Science Solutions) in combination with a UPLSAPO 60× oil immersion objective (NA:1.35) at 2× zoom, and stable cells were analyzed with the LSM 980 with Airy scan 2 (Zeiss, Jena) microscope. All images of transient cells were acquired and processed with the Olympus FluoView Software, whereas images of stable cells were acquired and analyzed with the Zeiss Zen 3.2 Blue edition software.

### Western Blot Analysis

For Western blot analysis, cells were cultured on 10-cm dishes as described above, washed once with ice-cold 1× PBS, and scraped using 1 ml of ice-cold 1× PBS. Collected cell suspensions were centrifuged at 300*g* for 10 min at 4 °C, and the supernatant was removed. Cell pellets were resuspended in RIPA lysis buffer (50 mM Tris-HCl pH 8.0, 150 mM NaCl, 1 mM EDTA, 0.1% SDS, 1% Triton-X-100, and 1× complete Protease inhibitor cocktail (Catalog No. 11836145001, Sigma-Aldrich), sonicated three times for 15 s with a Sonoplus sonicator (Catalog no. UW 2200 D-12207, Bandelin) with 0.8 amplitude, and centrifuged at 20,000 *g* for 10 min at 4 °C. The supernatant was collected and the protein concentration determined using the BCA protein assay (Catalog no. 23225, Thermo Fisher Scientific). Protein concentrations were adjusted to 2 μg/μl, samples combined with Laemmli buffer (46) (1× final concentration) and incubated for 1 hour at 37 °C. Proteins were separated by SDS-PAGE and transferred to nitrocellulose or PVDF membranes using semidry blotting. Subsequently, membranes were blocked in 5% skim milk dissolved in TBS-T (0.01% Tween 20) and incubated with the primary antibody overnight at 4 °C. The following primary antibodies were used, for experimental details see supplemental Table S14: HA (Catalog no. 12158167001, 3F10, Sigma-Aldrich), *GAPDH* (Catalog no. 32233, 6C5, Santa Cruz), *SDHA* (Catalog no. 14865-1-AP, Proteintech), *Calnexin* (Catalog no. 66903-1-AP, Proteintech), *GM130* (Catalog no. 610822, BD), *CTSD* (Catalog no. ab97499, Abcam), *NPC2* (Catalog No. 19888-1-AP, Proteintech), *LIMP2* (Catalog No. AF1966, R&D), *ATP6V1B2* (Catalog no. sc-166045, Santa Cruz), *ATP6V1D* (Catalog no. 14920-1-AP, Proteintech), *HGSNAT* (Catalog no. HPA029578, Sigma-Aldrich), *GBA* (Catalog no. 27972-1-AP, Proteintech), *RAPTOR* (Catalog no. 2280, Cell Signalling), *MAN2B2* (Catalog no. 17697-1-AP, Proteintech), *NEDD4* (Catalog no. 21698-1-AP, 1:1000), *CSTB* (Catalog no. sc-6493, Santa Cruz), *KIF5B* (Catalog no. ab 167429, Abcam), *FLOT1* (Catalog no. 15571-1-AP, Proteintech), *PPT1* (Catalog no. HPA021546, ATLAS), and β-actin (Catalog no. A5316, Sigma-Aldrich). The next day, blots were washed three times with TBS-T and incubated for 1 h at RT with the respective secondary antibodies (for details see supplemental Table S14): goat anti-mouse (Catalog no. 115035044, Dianova), goat anti-rabbit (Catalog no. 111035003, Dianova), or donkey anti-goat (Catalog no. 705035147, Dianova). Signals were detected using the enhanced chemiluminescence ECL kit (Catalog no. 1705061, Bio-Rad), visualized with the FUSION SOLO 4M System, and analyzed by the FUSIONcapt Advance Software (both PEQlab Biotechnology).

## Results

### Lysosomal Stability and Recovery Vary Between Cell Lines

For lysosome enrichment by SPIONs, it is crucial that the nanoparticles added to the cell culture medium are delivered to lysosomes through the endocytic route. In our hands, individual cell lines required distinct conditions to allow for optimal results. Therefore, we initially established the lysosome enrichment parameters for HEK293, HeLa, HuH-7, SH-SY5Y, MEF, and NIH3T3 cells. We chose the SPIONs approach over others, as it allows for the most efficient enrichment of large amounts of lysosomes with reduced loss of lysosome-associated complexes (18). For each cell line, we adapted conditions for coating of plates, density/number of cells, FCS content, growth time, and time points of SPIONs addition in order to achieve optimal results (for details see supplemental Table S1).

**Fig. 1:**
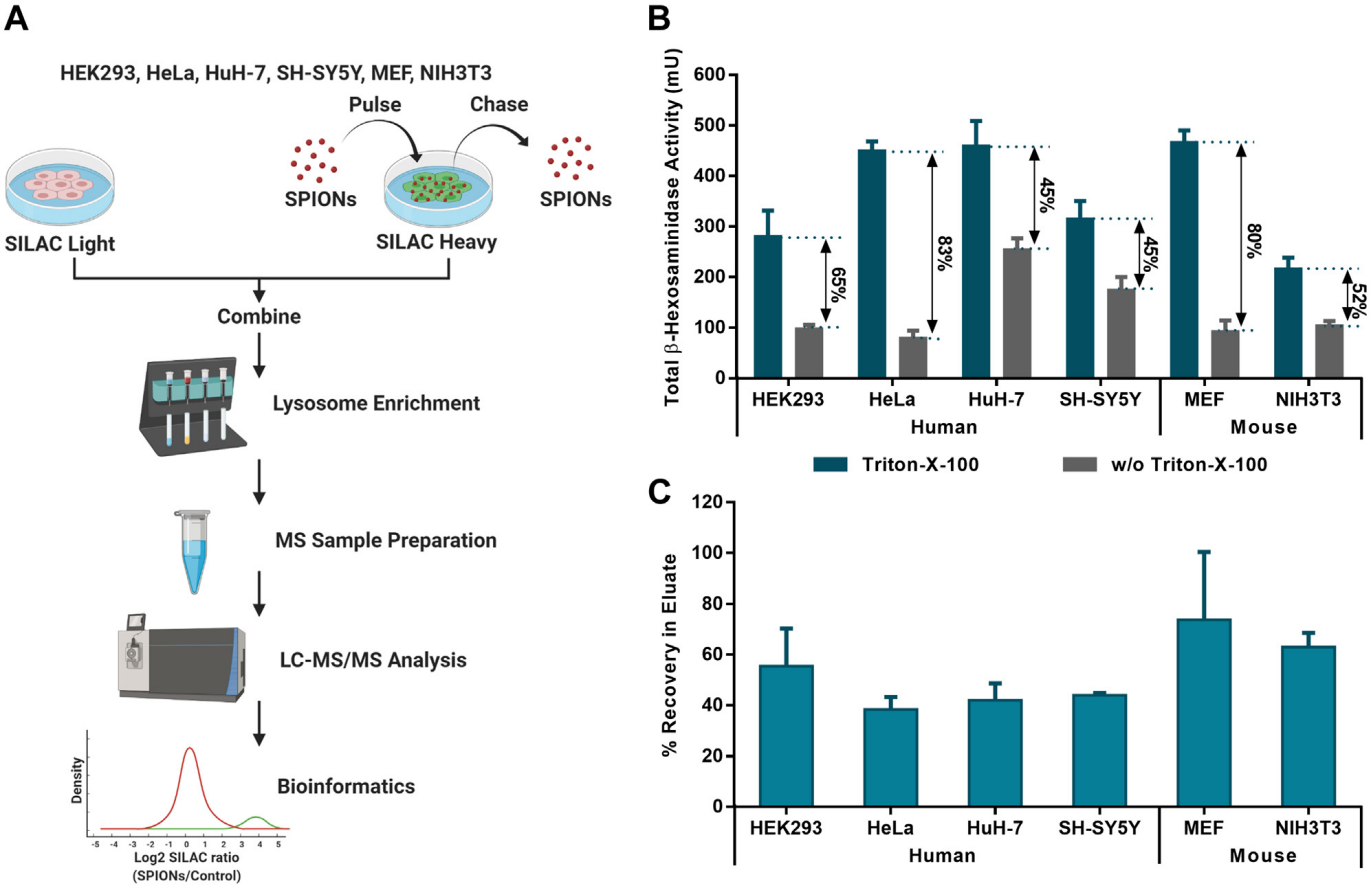
**Lysosomal stability and recovery vary across different cell types.***A*, workflow for lysosome enrichment and mass spectrometric analysis. *B*, activity of the lysosomal luminal enzyme β-hexosaminidase determined from postnuclear supernatant fractions of combined light/heavy SILAC cells. β-Hexosaminidase activity with/without the addition of Triton-X-100 relates to the total fraction of lysosomes contained in the sample and those that ruptured during cell lysis, respectively. *C*, lysosome recovery rates in input and eluate fractions of lysosome enrichment experiments after correction for the presence of background cells not receiving SPIONs determined by β-hexosaminidase activities. Shown are mean values ±SD, n = 4. LC-MS/MS, liquid chromatography–tandem mass spectrometry; MS, mass spectrometry; SILAC, stable isotope labeling by amino acids in cell culture; SPION, superparamagnetic iron oxide nanoparticle.

The enrichment of lysosomes from mammalian cells typically results in high numbers of contaminating nonlysosomal proteins (23). In order to be able to discriminate between lysosome-specific proteins and such binding to the column in an unspecific way, we included differentially SILAC-labeled control cells, which did not receive SPIONs, acting as an internal standard. The two populations of cells were combined in equal numbers before lysis. In this setting, the likelihood of retention on the magnetic column for unspecifically binding proteins is irrespective of the presence of SPIONs containing lysosomes. Therefore, for SPIONs (S)-treated and control (C) samples, these proteins should be detected with a ratio of S/C = 1. However, proteins that are localized at the lysosome, and therefore enriched in a specific way, should be detected with a ratio of S/C > 1 (Fig. 1*A*).

Following this strategy, we performed four biological replicates for each cell line. In order to account for systematic errors related to SILAC labeling and data analysis, we performed label switching, including SPIONs in two replicates of light and heavy labeled cells, respectively. After pooling and disruption of cells, we assessed lysosomal integrity using activity assays for the lysosomal luminal enzyme β-hexosaminidase (Figs. 1*B* and S1 (19)) and the purity of our lysosome-enriched fractions by Western blot analysis for marker proteins of lysosomes, mitochondria, the endoplasmic reticulum (ER), and the Golgi apparatus (supplemental Fig. S2). The highest total enzymatic activity was determined for MEF, HeLa, and HuH-7 cells, indicating that they contain most lysosomes. We observed the highest percentages of intact lysosomes for MEF and HeLa cells (fraction of intact lysosomes of >80%), while HuH-7 and SH-SY5Y cells revealed the highest lability/organelle disruption (∼45% intact lysosomes). For the normalized recovery of intact lysosomes, MEFs performed best, while, surprisingly, HeLa cells yielded the lowest amount (∼2-fold difference, Figs. 1*C* and S1). The fact that lysosomes from HeLa cells showed a very good intact ratio and the β-hexosaminidase activity from their input fractions were among the highest of all cell lines (supplemental Fig. S1), but the yield was lowest (Fig. 1*C*), indicates that the percentage of lysosomes receiving SPIONs through unspecific fluid phase endocytosis in these cells is lower in comparison with the other lines.

### Proteomic Analysis of Lysosome-Enriched Fractions Identifies Unique Expression Patterns in Individual Cell Lines

**Fig. 2:**
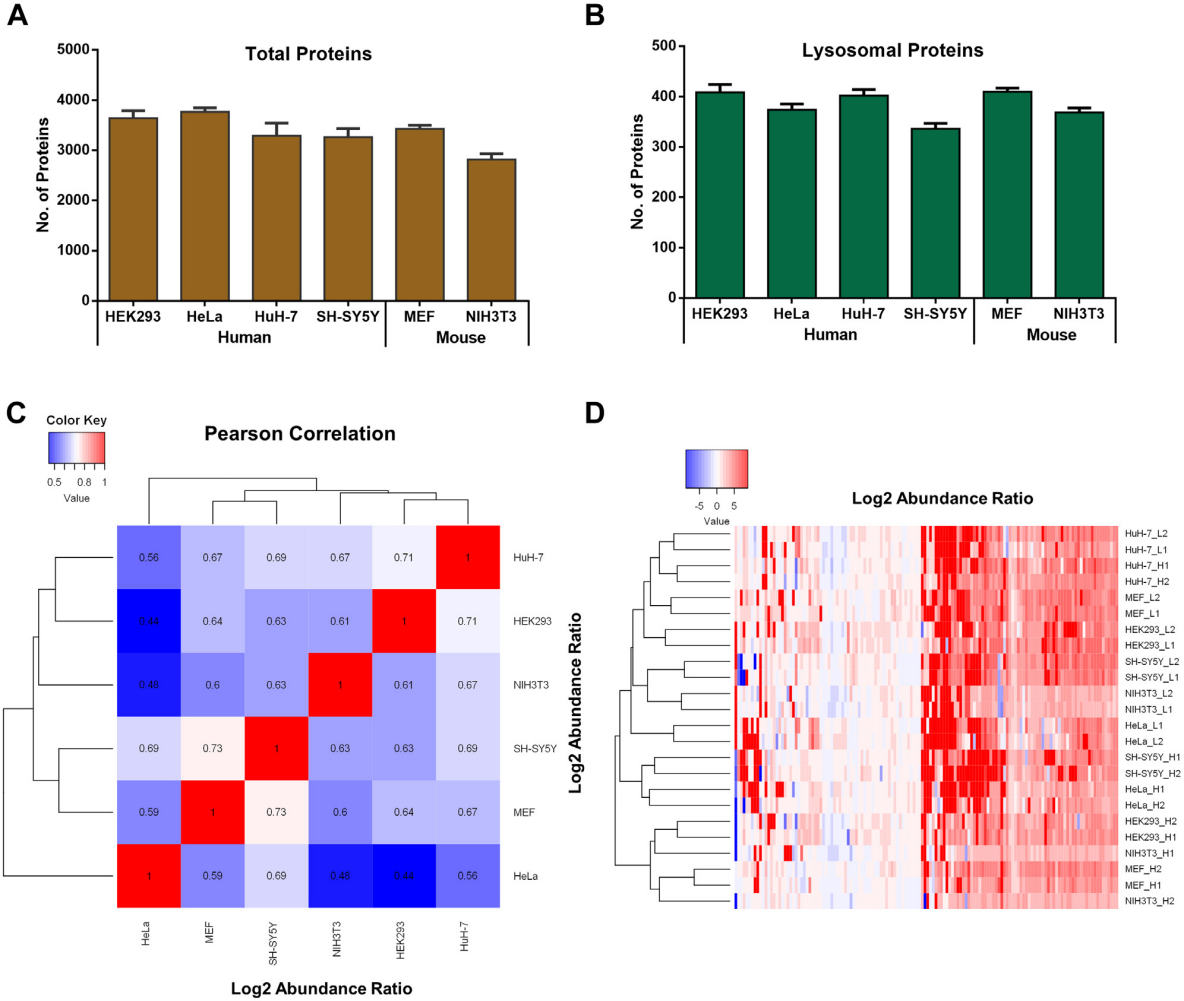
**Proteomic analysis of lysosome-enriched fractions identifies unique patterns of individual cell lines.***A*, average number of identified proteins detected for individual cell types. *B*, average number of identified putative lysosomal proteins detected for individual cell types. *C*, Pearson correlation values of log2 abundance ratios (SPIONs/control) for putative lysosomal proteins across individual cell lines. *D*, heatmap and unsupervised hierarchical clustering of log2 abundance ratios (SPIONs/control) for putative lysosomal proteins. Shown are mean values ±SD, n = 4. SPION, superparamagnetic iron oxide nanoparticle.

Using an optimized protocol (23), we analyzed the lysosome-enriched fractions of the individual cell lines by mass spectrometry–based proteomics (Fig. 1*A* and supplemental Table S2). After filtering for peptide and protein identifications with a FDR of 1%, we identified 8237 proteins from >1,000,000 peptide spectral matches across all cell lines. While the four human cell lines contributed 7289 unique identifications in total (63%–73% detected in each cell line), 5235 were identified in the two mouse cell lines with 75% and 90% unique identifications, respectively (Figs. 2*A* and S3*A* and supplemental Table S2). We observed the highest and lowest average numbers of total proteins identified per replicate for HeLa and NIH3T3 cells, respectively, while, when considering only proteins that were covered reproducibly in all four replicates, HeLa, HEK293, and MEF cells performed best (supplemental Fig. S3*C*). As we were especially interested in proteins located at the lysosome, we subsequently extracted a subset of proteins that are assigned to the lysosomal compartment in PANTHER and/or UniProt for mouse or human, irrespective of the fact if they fulfil a crucial function for this organelle (supplemental Table S2). For these putative lysosomal proteins, samples derived from HEK293, HuH-7, and MEF cells resulted in the highest average number of identifications for individual runs, while SH-SY5Y cells performed worst (Figs. 2*B* and S3, *B* and *D*).

To investigate the correlation among individual cell lines, we determined the Pearson correlation coefficients for the log2 abundance ratios of the identified proteins. On the whole protein level, NIH3T3 cells showed the lowest correlation with all other cell lines, while SH-SY5Y cells were most similar (supplemental Fig. S3*E*). When considering only the putative lysosomal proteins, correlation coefficients were in general higher. The exceptions were comparisons of HeLa and NIH3T3/HEK293 cells, yielding the lowest values, indicating stronger quantitative differences between the lysosomal proteomes of these cell lines (Fig. 2*C*). Based on unsupervised hierarchical clustering, we observed a general clustering for samples that received SPIONs in the light or heavy SILAC channel, respectively (supplemental Fig. S3*F*). Since especially unspecific binding proteins are affected by the SILAC label switch, this observation underlines the strong contribution of background proteins to the individual datasets. Also for putative lysosomal proteins, we observed an effect of the SILAC labeling state of the cell line, but no general clustering occurred depending on which SILAC labeled cell line received SPIONs (Fig. 2*D*).

### Putative Lysosomal Proteins are Identified with a Higher Reproducibility Between Different Cell Lines

Based on the heterogeneity of tissue-specific phenotypes for individual lysosomal storage disorders (47), it is highly likely that lysosomes in distinct cell types exhibit unique properties. While studies investigating individual lysosomal proteins by, *e.g.*, Western blot, quantitative PCR, or immunostaining approaches have shown highly variable expression levels across tissues, to our knowledge so far no attempts have been made to compare expression levels of lysosomal proteins between cell lines on a global scale. It remains therefore largely elusive in how far lysosomes differ between cell types.

**Fig. 3:**
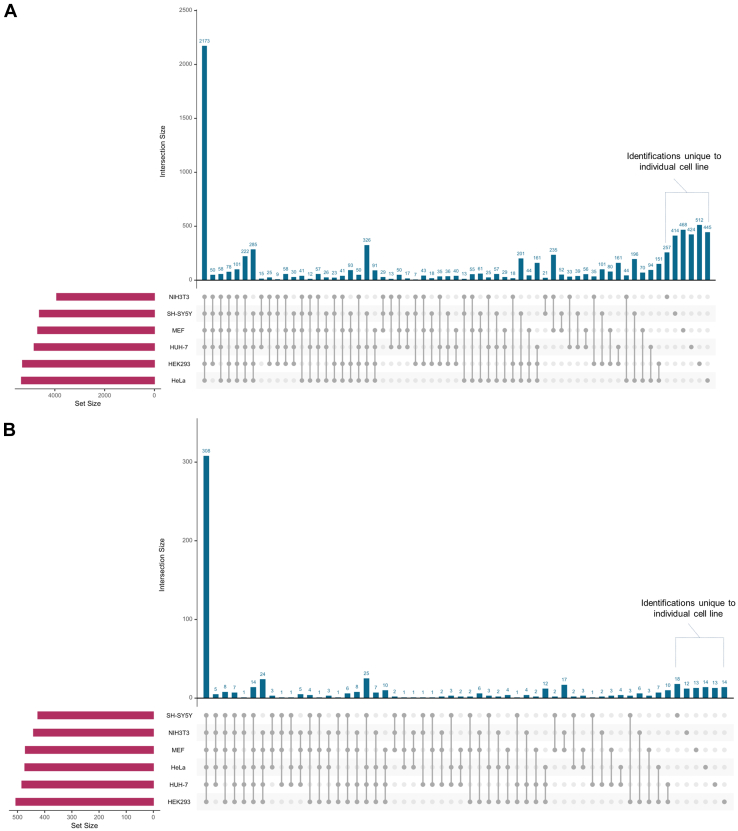
**Proteins with a putative lysosomal localization are identified with a higher reproducibility between individual cell lines.** Dataset size as well as individual overlaps for distinct combinations are indicated. *A*, all proteins identified in the lysosome-enriched fractions of the individual cell types. *B*, putative lysosomal proteins identified in lysosome-enriched fractions of the individual cell lines.

To further assess differences in the lysosomal proteomes of the individual cell lines analyzed, we performed direct comparisons of the identified proteins (Fig. 3 and supplemental Table S3). Of the 8704 proteins assigned in total, only 2173 proteins were found in all six cell lines, while 2520 were unique to one of them (Fig. 3*A*). HEK293 and NIH3T3 cells contributed with 512 and 257 proteins, respectively, the highest and lowest numbers of unique identifications. In all four human cell lines, 326 proteins were identified that were not detected in the two mouse lines, while 235 were unique for the mouse cells.

For the putative lysosomal proteins identified across all datasets (643 in total), we observed a similar behavior, however, with a markedly increased reproducibility compared with the whole dataset (48% of lysosomal proteins *versus* 25% of total proteins were identified in all six cell lines, Fig. 3*B*). Furthermore, we detected 130 lysosomal proteins that were unique to human cells (25 reproducibly detected in all four lines), while 42 proteins were unique to mouse cells (17 detected in both cell lines). Overall, while the majority of proteins were reproducibly detected across cell lines, each comparison of the individual proteomes resulted in unique subpopulations. This indicates distinct features of the lysosomes of the respective cells, which are possibly related to their individual functions/characteristics.

### Intensity-Based Absolute Quantification Allows for Estimation of Abundance Levels for Known Lysosomal Proteins

In mass spectrometry experiments, also low abundant proteins are often reproducibly detected, if the sample complexity does not exceed the analytical setup’s limitations defined by the instrument’s sensitivity and speed, as well as the chromatographic gradient length. Therefore, protein identification does not necessarily correlate with abundance, and differences in identification frequently only reveal extreme cases of expression variability, possibly underestimating the extent of (lysosomal) heterogeneity. Furthermore, every cell requires a certain lysosomal “core proteome” to be able to deal with the turnover of ubiquitous cellular components. We argued that it is therefore highly unlikely that proteins belonging to this class are not expressed at all. It should be more reasonable that the expression levels of individual proteins belonging to this group are adapted to the specific characteristics of an individual cell type, reflecting its unique composition and function (47).

To be able to address lysosomal heterogeneity based on changes in protein abundance in more detail, we investigated the expression levels for our list of putative lysosomal proteins (supplemental Table S2) across all cell lines. We utilized the intensity-based absolute quantification (iBAQ) value (supplemental Table S4), which is a measure for absolute protein quantity (48, 49), to be able to compare expression levels of human and mouse cells. As the yield of lysosomes varied among cell lines and replicates (supplemental Fig. S1), we corrected for inconsistencies by replicate-wise normalization of individual iBAQ values (supplemental Table S5). For this purpose, we utilized the median iBAQ value from eight core subunits of the V-ATPase complex (detected with ≥10 unique peptides each), which is responsible for lysosomal acidification (50). The abundance of these proteins was among the most reproducible when considering all lysosomal proteins in the datasets (supplemental Figs. S4*A* and S5*A*), presenting a “core feature” of lysosomes. This is probably also related to the fact that the presence and correct stoichiometry of the V-ATPase are essential for lysosomal function. Subsequently, we filtered for proteins with iBAQ values in at least two cell lines and three biological replicates each, calculated the median of the V-ATPase-normalized value for individual proteins, and grouped them into 32 classes based on their function and/or localization (supplemental Table S5).

### Expression Levels of Lysosomal Proteins Vary Between Lysosome-Enriched Fractions of Individual Cell Lines

Initially, we calculated the sum of V-ATPase-normalized iBAQ values in the individual cell lines to estimate how many lysosomal proteins are present relative to the V-ATPase (supplemental Fig. S4*B*). Lysosomes enriched from HuH-7 and HeLa cells contained the highest amounts (4- and 2.3-fold more than, *e.g.*, MEFs, respectively), while values in the other cell types were roughly similar. The observed differences were mainly due to the strong overrepresentation of hydrolases in HuH-7 cells, transporters in HeLa cells, and membrane proteins in both of them. Only lysosome-associated ubiquitin ligases and proteins related to *mTORC1*, which were most abundant in SH-SY5Y and HEK293 cells, respectively, were the highest in abundance in cell lines other than HeLa and HuH-7 (supplemental Fig. S4*B*).

**Fig. 4:**
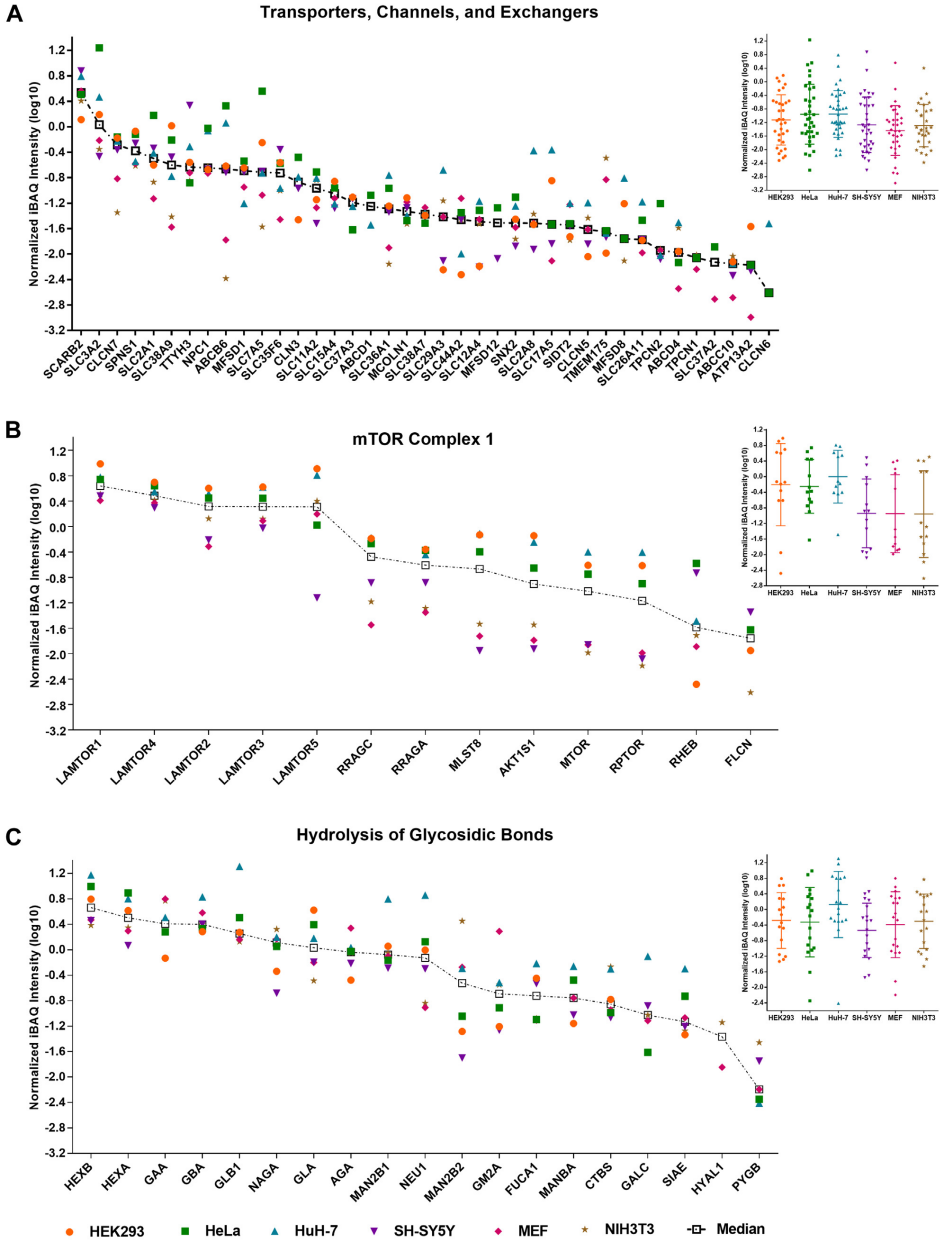
**Known lysosomal proteins show both highly conserved and diverse expression levels between individual cell lines.** For each protein, the median iBAQ values were determined and normalized to the median intensity of the same eight V-ATPase complex subunits in a replicate-wise manner. Proteins are either sorted based on their median intensity (scatter plot) or grouped in a cell line–wise manner (dotted box plot). *A*, proteins with known function as transporter, channel, or exchanger. *B*, members of, or proteins related to, mTORC1. *C*, proteins with a known function related to the hydrolysis of glycosidic bonds. Shown are log10 converted median-normalized iBAQ values for proteins detected in ≥3 replicates in each of ≥2 cell lines. iBAQ, intensity-based absolute quantification.

Subsequently, we investigated the expression levels of individual lysosomal proteins in the different cell lines. For the V-ATPase complex itself, we detected a conserved stoichiometry for proteins belonging to both the V0 and the V1 part with the exception of the associated proteins *TCIRG1* and *ATP6AP1/ATP6AP2* (supplemental Fig. S5*A*). For proteins involved in the translocation of small molecules across the lysosomal membrane (Fig. 4*A*), we observed a dynamic range of three orders of magnitude with the cholesterol transporter *SCARB2* and the Cl^-^/H^+^ exchanger *CLCN6* showing the highest and lowest median expression levels, respectively (difference of ∼1400 fold).

Within certain functionally related groups, we detected both highly conserved and highly variable expression patterns. The Ca^2+^ channels *MCOLN1, TPCN1*, and *TPCN2*, for example, were expressed at very similar levels in all cell lines, while the amino acid- and oligopeptide-transporting members of the solute carrier (SLC) family showed both similar and highly variable expression patterns. *SLC3A2* and *SLC7A5*, which are known to heterodimerize (51), exhibited the highest dynamic range of all proteins in this group with differences of up to 135-fold between the highest (HeLa) and lowest (NIH3T3) expressing cell line, respectively. On the other hand, the sodium- dependent amino acid transporter *SLC38A7*, which was shown to be essential for the extracellular protein–dependent growth of cancer cells (52), was the most stable with a dynamic range of protein expression of ∼2-fold. Another member of the SLC38-family, *SLC38A9*, which is essential for the amino acid–dependent activation of *mTORC1* (8), exhibited with up to ∼40-fold a substantially higher difference in expression between cell lines. It was expressed highest in HEK293 cells, correlating with the pattern we observed for other proteins related to *mTORC1* (Fig. 4*B*). Overall, amino acid/oligopeptide transporters were expressed especially by HeLa cells at high levels (33-fold higher summed signal intensity compared with NIH3T3 cells), implying a high importance of amino acids derived from lysosomal protein degradation for their metabolism. This is in line with studies reporting the dependence of cancer cell metabolism on amino acids originating from lysosomal degradation of proteins (52, 53).

For other proteins related to *mTORC1*, we detected varying patterns (Fig. 4*B*). The Ragulator members *LAMTOR1*, *LAMTOR2*, *LAMTOR3*, and *LAMTOR4* were similarly expressed in all cell lines, while *LAMTOR5* was ∼100-fold more abundant in HEK293 compared with SH-SY5Y cells. For all proteins of this group, we observed, in general, high expression levels in HEK293, HeLa, and HuH-7 cells, with the exception of *RHEB*, which showed for HEK293 cells an inverse behavior compared with *SLC38A9*. In accordance with the role of *FLCN* in the *mTORC1*-dependent phosphorylation and cytosolic retention of the lysosomal transcription factors *TFEB* and *TFE3* (54, 55), we either detected *FLCN* (all cell lines except MEFs) or *TFEB/TFE3* (only MEFs) in the lysosome-enriched fractions.

For membrane and membrane-associated proteins, we observed a similar dynamic range as for transporters (supplemental Fig. S5*B*) and the highest abundances for *LAMP1* and *LAMP2*. Proteins involved in lysosomal positioning (mainly components of the *BORC* complex (56)) were present in similar abundances within the respective cell lines, with the exception of *ARL8B* and *DTNBP1* (supplemental Fig. S5*C*). For lysosome-associated ubiquitin ligases, on the other hand, no trend with respect to certain cell lines was detectable (supplemental Fig. S5*D*). For hydrolases, the dynamic range of expression was in general smaller than for proteins located at/in the lysosomal membrane, while proteins related to lipid metabolism (supplemental Fig. S5*E*) were less variable compared to proteases (supplemental Fig. S5*F*) and those involved in the hydrolysis of glycosidic bonds (Figs. 4*C* and S5*G*). For the latter, we observed, for example, a highly conserved abundance across cell lines for *GBA*, while other proteins, such as *NEU1* or *MAN2B2*, presented with variabilities of up to ∼140-fold. For the majority of hydrolases, HuH-7 cells showed the highest expression levels, indicating a higher throughput of substrates in their lysosomes, which is probably related to the liver’s function as the main recycling organ of the body.

### Correlation of Lysosomal Protein Expression With Whole Cell Lysates Identifies Variability of Protein Expression and Distribution

**Fig. 5:**
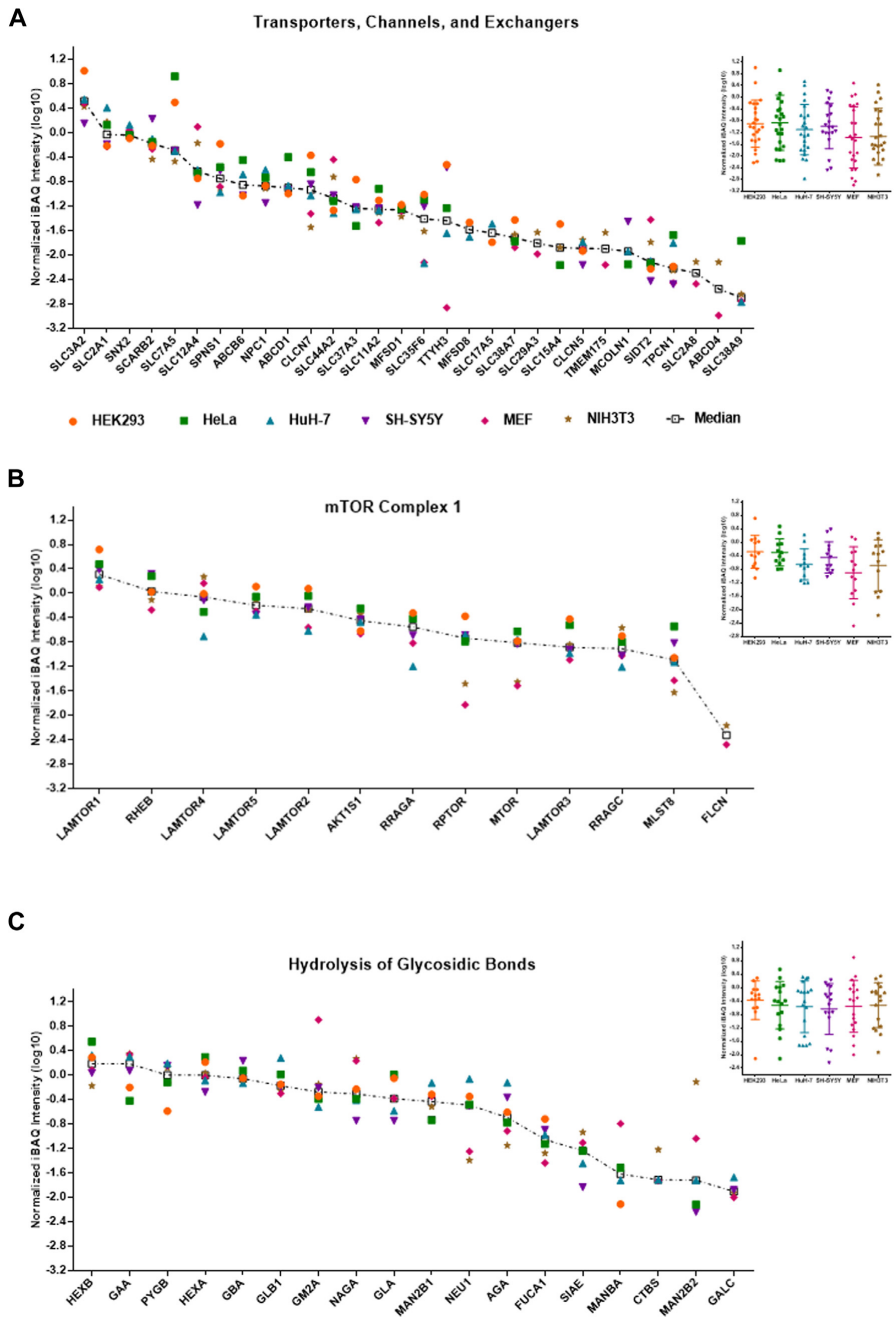
**Investigation of lysosomal proteins expression levels in whole cell lysate datasets of individual cell lines.** For each protein, the median iBAQ values were determined and normalized to the median intensity of the same eight V-ATPase complex subunits utilized for the lysosome-enriched fractions in a replicate-wise manner. Proteins are either sorted based on their median intensity (scatter plot) or grouped in a cell line–wise manner (dotted box plot). *A*, proteins with known function as transporter, channel, or exchanger. *B*, members of, or proteins related to mTORC1. *C*, proteins with a known function related to the hydrolysis of glycosidic bonds. Shown are log10-converted median-normalized iBAQ values for proteins detected in three replicates in each of ≥2 cell lines. iBAQ, intensity-based absolute quantification.

The observed differences in (lysosomal) protein abundance could originate from differential protein expression, differential association/localization with lysosomes, and/or variabilities in lysosome enrichment efficiency. We, therefore, further investigated lysosomal protein abundance in whole cell lysates from all six cell lines to investigate which of these possibilities are most likely (supplemental Fig. S6 and supplemental Table S6). We utilized DIA, as this analysis mode allows for investigation of most lysosomal proteins also from whole cell lysates (18, 57). Of 9107 proteins quantified across all cell lines, we were able to obtain values between 357 and 375 lysosomal proteins (supplemental Table S6). Subsequently, we calculated the V-ATPase-normalized iBAQ values in order to allow direct comparison with our lysosome-enriched sample dataset (Figs. 5 and S6 and supplemental Table S7).

In general, we observed a similar range of dynamic protein expression within individual cell lines, spanning >3 orders of magnitude between the lowest and highest expressed lysosomal proteins. When considering variability of expression levels for individual proteins across the six cell lines, we observed less pronounced differences (Figs. 5 and S6). This could be due to ratio compression effects, as the DIA-based quantification of low abundant lysosomal proteins fails to properly reflect protein levels in complex samples (57); nonexclusive lysosomal localization of individual proteins; or technical issues related to lysosome enrichment.

We, therefore, correlated signal intensities for proteins identified in both lysosome-enriched samples and whole cell lysates (supplemental Tables S5 and S7). We further investigated three functional groups of lysosomal proteins between whole cell lysates and lysosome-enriched samples, covering such located in its lumen (proteases and hydrolases of glycosidic bonds) and membrane (transporters, channels, and exchangers) (supplemental Fig. S7). For the majority of proteins, we identified similar trends for the relation of signal intensities of lysosome-enriched samples and whole cell lysates. This indicates a good representation of the cellular pool of lysosomal proteins in our lysosome-enriched datasets as, especially for the luminal proteins, recovery of intact lysosomes is a prerequisite for their enrichment. Even though we utilized V-ATPase-normalized values for both datasets, intensities in the lysosome-enriched fractions were higher for most hydrolases (supplemental Fig. S7, *A* and *B*). This could be related to signal suppression effects for peptides originating from low abundant lysosomal proteins in highly complex samples (57), which is also in line with the fact that we were not able to quantify all proteins in whole cell lysates for which we obtained robust values in lysosome-enriched samples.

Interestingly, we identified both for luminal hydrolases and (to a higher extent) for transporters and exchangers, also proteins that were of higher relative abundance in whole cell lysates (for individual proteins and values see supplemental Table S7). This indicates that the cellular pool of certain lysosomal proteins is not completely present at this organelle but only a subfraction is located there. While this was in all cell lines only the case for a few hydrolases, more transporters, channels, and exchangers behaved this way.

In order to further follow up on the heterogeneity of lysosomal protein expression and distribution, we validated the abundance of selected lysosomal or lysosome-associated proteins by Western blotting in whole cell lysates and lysosome-enriched fractions from all six cell lines. Based on the quantification of individual Western blot bands, we observed matching trends of protein expression for 80% of intensities in whole cell lysates and 85% of intensities in lysosome-enriched fractions (supplemental Fig. S8 and supplemental Table S14). This further confirms that the expression/distribution of individual lysosomal proteins can vary between the investigated cell lines.

### Bimodal Distribution Analysis Revealed Specifically Enriched Proteins

In experiments dealing with the analysis of lysosome-enriched fractions by LC-MS/MS, significantly more proteins are identified than those likely to be located at the lysosome, irrespective of the enrichment method or analytical strategy (18, 20, 23). Also, in our dataset, we identified for each individual cell line >4000 unique proteins in the lysosome-enriched fraction. Based on the putative list of lysosomal proteins (supplemental Table S2), and GO analyses of the respective datasets (18, 44, 58), it can be reasonably assumed that a large portion of these proteins is retained at the column material due to unspecific binding to the stationary phase, representing contaminations, or to structures interacting with lysosomes (such as the cytoskeleton or the ER), which result in coenrichment of other (non-lysosome-specific) proteins interacting with them. Therefore, it is difficult to identify novel lysosomal proteins, and studies dealing with the analysis of lysosome-enriched fractions by mass spectrometry–based proteomics often do not attempt to infer lysosomal localization from identification but rather focus on known lysosomal proteins contained in the dataset (27, 28).

**Fig. 6:**
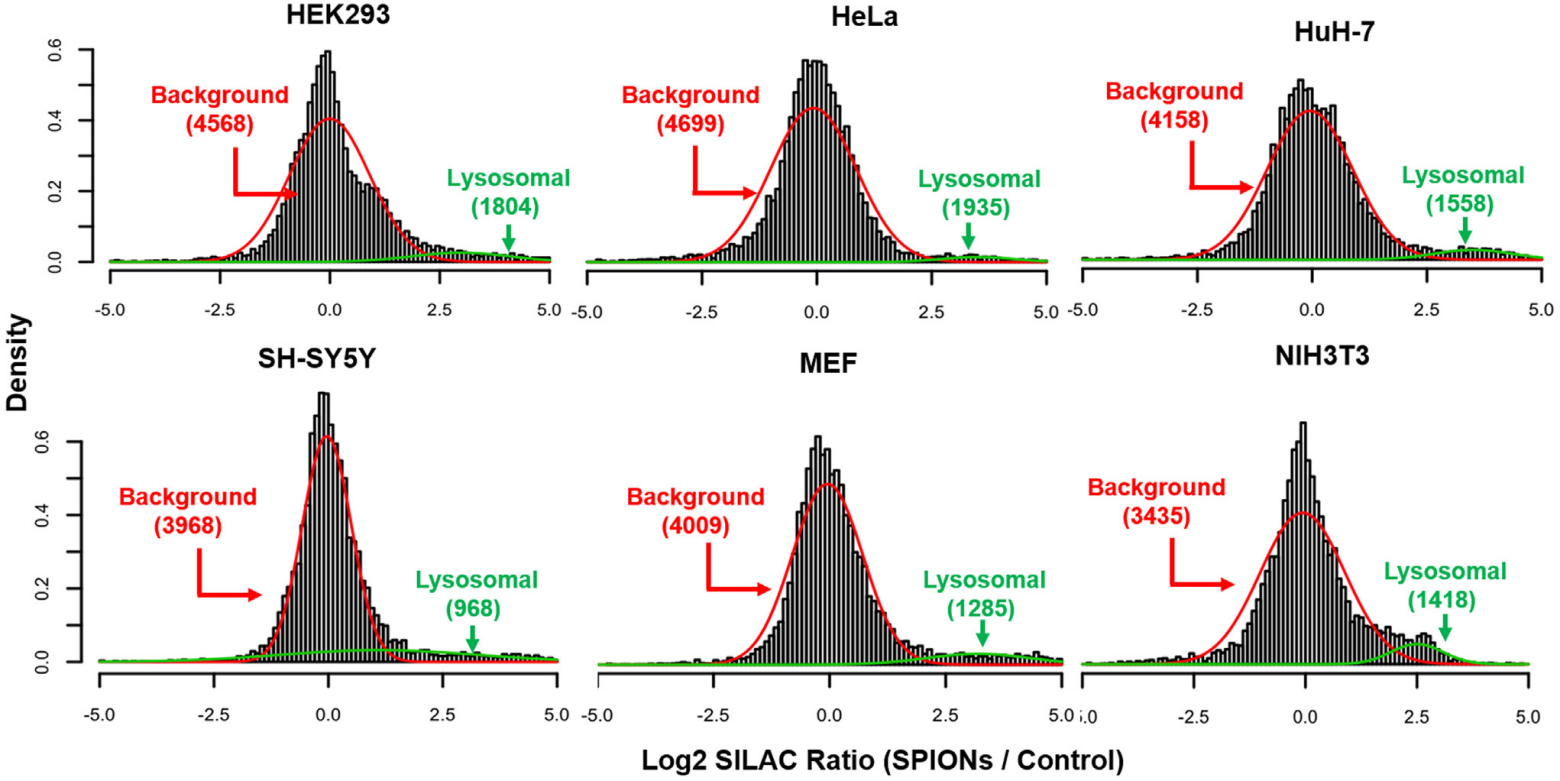
**Bimodal distribution analysis of differentially SILAC-labeled populations of SPIONs-receiving and control cells identifies potential lysosomal proteins.** Histograms indicate binned frequencies of log2-transformed normalized SILAC ratios across the datasets. Normal distributed populations were calculated using an expectation-maximization algorithm, and a *p*-value of ≤0.05 was applied as cutoff. Red lines indicate the background population showing a similar behavior between control cells and such receiving SPIONs. Green lines indicate proteins with a significant difference in their SILAC ratio for cells receiving SPIONs relative to the background population. SILAC, stable isotope labeling by amino acids in cell culture; SPION, superparamagnetic iron oxide nanoparticle.

We argued that inclusion of a population of control cells, which did not receive SPIONs, should allow for discrimination of lysosome-specific and background proteins, as the chance of enrichment for unspecific interactors should be irrespective of the presence of SPIONs in the sample. Therefore, in all lysosome enrichment experiments, we combined SPIONs-receiving cells with a differentially SILAC-labeled population of untreated “background cells” (Fig. 1*A*). We calculated the log2-transformed ratios of SPIONs/control, median-normalized the values for individual replicates to compensate for differences in lysosome enrichment efficiency, and performed a bimodal distribution analysis. For each cell line, we estimated the mixture of two univariate normal distributions and assigned a posterior probability of lysosomal localization to each protein using an expectation-maximization algorithm (40). This resulted in the identification of two overlapping normal distributions presenting on the one hand the “background population” of proteins, which bind unspecifically to the beads, and on the other hand the “lysosomal population” of proteins, showing increased binding due to the presence of SPIONs in the cells’ lysosomes (Fig. 6 and supplemental Table S8). Based on these distributions, we applied an adjusted *p*-value cutoff of <0.05 for the definition of enrichment by SPIONs. This resulted for each cell line in a high confidence subpopulation of proteins identified in the individual lysosome-enriched fractions, encompassing 25% to 41% of the respective datasets (supplemental Table S9).

### Identification Frequency Correlation Determines High Confidence Lysosomal Proteins

**Fig. 7:**
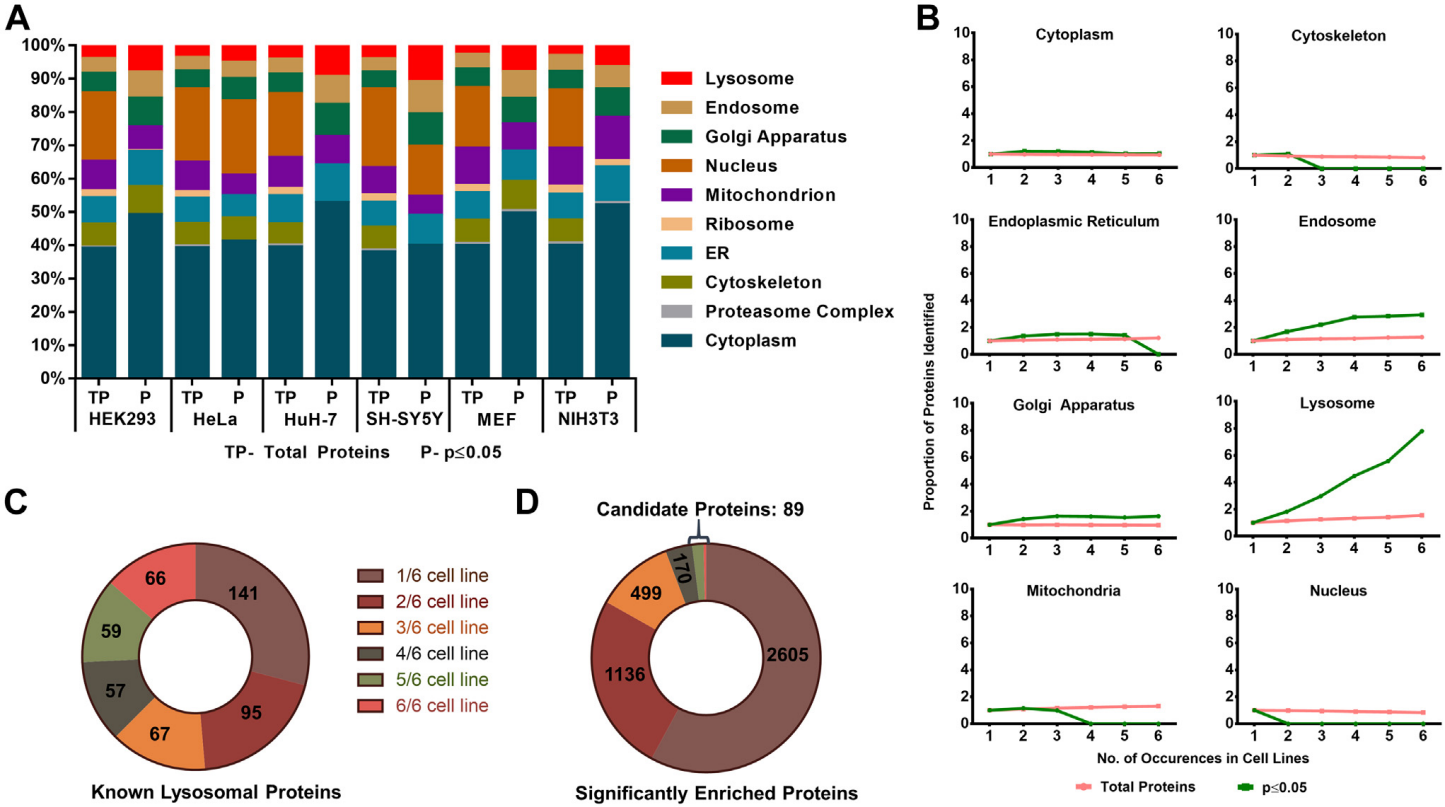
**Frequency of identification across cell lines correlates with lysosomal localization and allows for identification of high confidence novel lysosomal proteins.***A*, gene ontology (GO) analysis of all proteins contained in the respective datasets (total proteins, TP) and proteins that were determined to be significantly overrepresented in SPIONs-receiving cells (based on bimodal distribution analysis, *p*-value ≤0.05). The percentage of proteins (relative to the respective dataset) is shown based on their assignment to significantly enriched GO terms (FDR <0.05, Fisher's test). *B*, correlation of identification frequency and GO term distribution for total proteins and such overrepresented in SPIONs-receiving cells. Shown values represent the percentage of proteins assigned to a respective category normalized to the value for considering presence in at least one cell line. *C*, distribution of putative lysosomal proteins depending on their identification frequency. *D*, distribution of proteins determined to be specifically enriched by SPIONs in bimodal distribution analyses excluding putative lysosomal proteins depending on their identification frequency.

In order to assess the effect of our bimodal distribution analysis on the individual datasets, we performed GO analyses for all proteins of each dataset, as well as for the subpopulations of high confidence SPIONs-enriched proteins, using the PANTHER overrepresentation test (44, 58). While the proteins in our datasets were assigned to 10 cellular compartments before the bimodal distribution analysis, we were able to entirely deplete certain categories—presenting presumably nonspecifically enriched proteins—for several cell lines (Fig. 7*A* and supplemental Table S10). For example, in HEK293, HuH-7, MEF, and NIH3T3 cells, all nuclear proteins were excluded from the dataset. This was, however, not the case for HeLa and SH-SY5Y cells, despite the fact that total numbers could be reduced by 63% and 86%, respectively (supplemental Table S10). We observed a similar effect for proteins related to the cytoskeleton, proteasome, ribosomes, and mitochondria, which were depleted from several cell lines. Most importantly, we were able to achieve an increase for the percentage of putative lysosomal proteins in all cell lines, ranging up to 3-fold (in the case of MEFs). This confirms the capability of the approach to enrich the dataset for proteins located at the lysosome. For proteins assigned to endosomes, Golgi apparatus, and ER, however, only a fraction was removed. Furthermore, compared with the total dataset, the relative abundance of certain contaminating categories further increased in the high confidence SPIONs enriched list of proteins. This is due to the reduction in size of the total protein population considered for the analysis, as the number of proteins assigned to these contaminating categories was significantly reduced.

Despite the increase in abundance for putative lysosomal proteins, their percentage in relation to the whole dataset peaked at only 11% (SH-SY5Y cells). As we detected putative lysosomal proteins more reproducibly across the individual cell lines (Fig. 3*B*), we hypothesized that the frequency of identification of an individual protein in multiple cell lines could be used as an indicator for the likelihood of lysosomal localization. We tested this hypothesis by correlation of the percentage of proteins belonging to a certain GO category with their frequency of identification across the individual datasets of the six cell lines (Fig. 7*B* and supplemental Table S11). For the whole datasets, we did not observe any correlation of reproducibility with the abundance of a certain category, with the exception of ribosomal proteins, whose percentage increased by 2-fold when comparing proteins identified in 6/6 cell lines with those identified in ≥1 cell line (supplemental Table S11). When we considered our list of *p*-value filtered high confidence lysosomal proteins, however, we observed a striking increase in the enrichment of lysosomal proteins by up to ∼8-fold (proteins identified 6/6 *versus* ≥ 1 cell lines, Fig. 7*B*). At the same time, we were able to deplete proteins from the cytoskeleton, nucleus, and mitochondria, while the percentage of cytoplasmic and Golgi apparatus proteins remained stable. For proteins assigned to endosomes, we observed the highest increase (∼3-fold) after such of lysosomal origin, indicating a specific enrichment, probably due to SPIONs, which did not completely transfer to the lysosomal compartment during the chase period. The increase in percentage of lysosomal proteins relative to the whole dataset confirmed that true positive lysosomal proteins are more likely to be reproducibly detected across several cell lines.

Next, we compared our dataset with results from previously published studies that identified potentially novel lysosomal proteins with other approaches. We considered datasets based on proximity biotinylation with BirA∗ (59), density gradient centrifugation (24, 60, 61), enrichment of mannose 6-phosphate–modified proteins (62), and immunoprecipitation of lysosomes through a 3× HA-tagged version of the lysosomal membrane protein TMEM192 (20). When we compared the individual published datasets with our list of proteins, considering such which were significantly enriched in our bimodal distribution analysis in at least one of the cell lines, we observed an overlap of 33%–70% (supplemental Fig. S9 and supplemental Table S12). When we compared the combined proteins of all published datasets with our data (proteins identified in at least one cell line), we observed an overlap of 961 potential lysosomal proteins. Strikingly, these data revealed a clear correlation of the identification frequency across our six cell lines with the chance to be detected in one of the other datasets: of the proteins identified in 6/6 and 5/6 cell lines in our study, 91% and 65% had already been found in the mass spectrometry–based analysis of lysosome-enriched fractions of the previous studies.

We further investigated the overlap of the putative lysosomal proteins reported in the literature and public repositories (supplemental Table S2) with those detected in the significantly enriched fractions of 5/6 and 6/6 cell lines. We found that 42% and 77% of these high confidence potential novel lysosomal proteins were shared between both lists (Fig. 7*C*). Finally, we removed previously reported putative lysosomal proteins from the high confidence lysosomal identifications in our dataset, resulting in a list of potentially novel lysosomal proteins. With respect to the complete list of proteins identified in at least one of the cell lines, only 2% were reproducibly identified in ≥5 cell lines, presenting high confidence potential novel lysosomal proteins (Fig. 7*D* and Table 1). Among these 89 proteins, GO analysis revealed an enrichment in membrane proteins and transporters (supplemental Fig. S10 and supplemental Table S13).

**Table 1:** Potential novel lysosomal proteins based on their overrepresentation in SPIONs-receiving cells for ≥5 cell lines

Candidates evaluated by immunostaining are highlighted. ∗*TSPAN3* was identified in six cell lines but only enriched in the SPIONs-receiving fraction of four cell lines.

### Confirmation of lysosomal Localization for Candidate proteins by Immunostaining

In order to confirm the validity of our approach, and to follow up on potentially novel lysosomal proteins, we selected six candidates that were enriched in 6, 5, or 4 cell lines (Table 1) for follow up studies. All of these proteins were also found previously in at least one other proteomic study to be enriched in the lysosomal fraction (supplemental Table S12). This provided further credibility to their potential lysosomal localization. For most of them, however, no additional proof existed to confirm their lysosomal localization, leaving the question open if they presented unspecifically enriched proteins or such of true lysosomal localization. Owing to the strong overrepresentation of membrane proteins in our list of high confidence candidates, we focused on proteins that are localized in or at the lysosomal membrane: 1. *TM7SF3* (Transmembrane 7 superfamily member 3), which was shown to maintain protein homeostasis through attenuation of ER stress (63); 2. *SLC12A9* (Solute carrier family 12 member 9), which belongs to the *SLC12* family of electroneutral transporters facilitating the symport of Na^+^/K^+^ with Cl^-^ (64); 3. *SLC31A1* (High affinity copper uptake protein 1), which belongs to the *SLC31* family of copper transporters (65); 4. *TMEM63B* (*CSC1*-like protein 2), an osmosensitive Ca^2+^ permeable channel (66); 5. *TSPAN3* (Tetraspanin-3), a member of the tetraspanin superfamily, which has been involved in trafficking of membrane proteins (67); and 6. *NDFIP2* (*NEDD4* family-interacting protein 2), which was reported to be involved in controlling the activity of WW-HECT domain E3 ubiquitin ligases, in particular *NEDD4* (68).

While only evidence from proteomic large-scale studies existed for the lysosomal localization of *TM7SF3*, *SLC12A9*, *TMEM63B*, and *TSPAN3* (41, 42), both *SLC31A1* and *NDFIP2* were found in previous studies to colocalize at least partially with lysosomes. *SLC31A1* was shown to be predominantly localized at the plasma membrane and also to colocalize with endosomes and/or lysosomes (61, 69, 70). *NDFIP2* was found to localize to vesicular structures in the cytoplasm, with a significant colocalization to such being positive for lysosomal markers (71, 72).

**Fig. 8:**
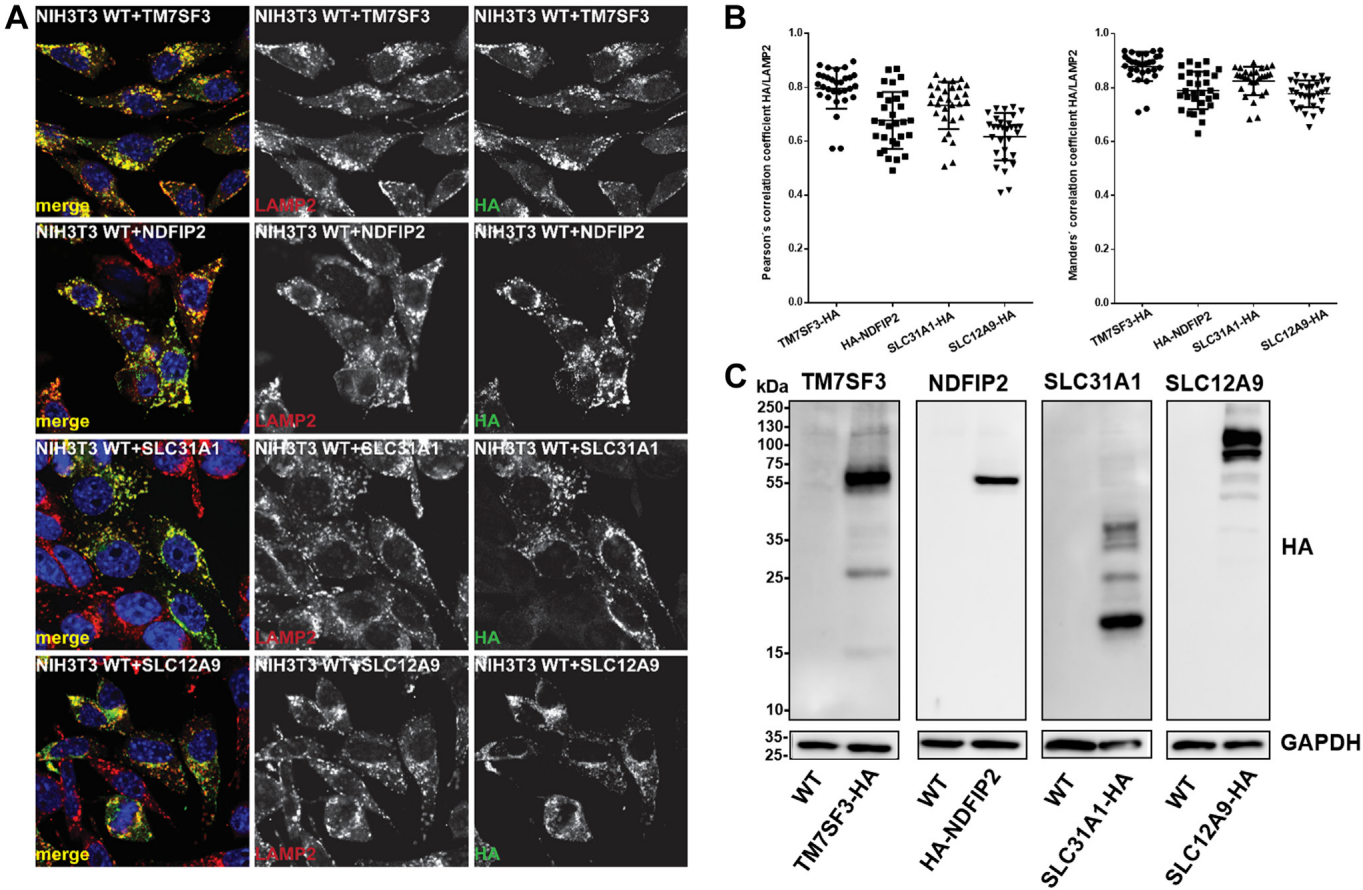
**Investigation of subcellular localization of lysosomal candidate proteins shows their predominantly lysosomal localization.** NIH3T3 cell lines stably transfected with N-/C-terminally HA-tagged constructs of *TM7SF3*, *NDFIP2*, *SLC31A1*, or *SLC12A9*, which were detected in the significantly enriched fractions (*p* ≤ 0.05) in at least five cell lines. *A*, colocalization with the lysosomal marker *LAMP2* investigated by immunostaining. *B*, Pearson's and Manders' correlation coefficients for the colocalization of signals for HA and *LAMP2* for individual cell lines. *C*, Western blot analysis of stable transfected and wildtype cells. Detection of construct with anti-HA; *GAPDH* serves as loading control.

For each candidate, we transiently transfected HeLa cells with an expression vector containing an HA- or MYC-tagged version of the respective cDNA and investigated lysosomal localization by coimmunostaining with an antibody directed against the respective tag and the lysosomal marker protein *LAMP2* (supplemental Fig. S11). In these experiments, five of our six candidate proteins (*TM7SF3, SLC12A9, SLC31A1, TMEM63B*, and *TSPAN3*) colocalized with *LAMP2*, thereby confirming their lysosomal localization. For *NDFIP2*, however, we only observed a partial colocalization with *LAMP2* while colocalization with the early endosomal marker *Rab5* was much more pronounced. Subsequently, we further generated stable NIH3T3 cell lines for *TM7SF3, NDFIP2, SLC31A1*, and *SLC12A9* and investigated these by coimmunostaining and Western blotting (Fig. 8). For all four candidate proteins, we observed a strong colocalization with the lysosomal marker *LAMP2* (Fig. 8*A*) with median Manders' coefficients between 0.7 and 0.8 and Pearson's coefficients between 0.6 and 0.8 (Fig. 8*B*), indicating their predominantly lysosomal localization. Based on these experiments, we were able to show lysosomal localization for all six candidates in cells either transiently or stably overexpressing the respective protein, presenting for four of them a first proof with a method orthogonal to large-scale proteomics studies.

## Discussion

This study presents a systematic comparison of lysosome-enriched fractions from different cell lines. Already during cell lysis and lysosome enrichment, differences between the individual cell types became apparent, as we observed varying yields of β-hexosaminidase activity and lysosomal intact ratios in the input fraction preceding lysosome enrichment, as well as different recovery rates in the eluate fraction (Fig. 1, *B* and *C*). Surprisingly, the V-ATPase-normalized iBAQ levels of both β-hexosaminidase subunits HEXA and HEXB (Fig. 5*C*) did not match the pattern of enzymatic activity for all cell types, as both proteins were significantly less abundant in MEF than in HeLa and HuH-7, which all showed similar enzymatic activity for whole cell lysates (Fig. 1*B*). This implies that MEF cells contain a higher number of lysosomes with lower individual HEXA/HEXB levels. This could be the reason that they performed best in terms of lysosome enrichment efficiency, as more lysosomes can receive SPIONs in these cells (Fig. 1*C*).

A possible factor for differences observed between individual cell lines could be the use of SPIONs, which consist of a dextran-coated iron oxide particle. It is currently debated in how far the iron oxide core of SPIONs could result in biological effects, but conclusive data are lacking (73). A conceivable consequence of the presence of SPIONs could be the formation of reactive oxygen species *via* Fenton reactions, which would result in increased levels of protein oxidation. Investigation of data published previously by our group (18), however, did not reveal such effects as levels of oxidized methionine in lysosomes enriched by SPIONs were not higher than those obtained for lysosomes enriched by immunoprecipitation of tagged lysosomal membrane proteins (18). Therefore, oxidation of lysosomal proteins by SPIONs seems unlikely, indicating effective shielding of the iron oxide core by the dextran coat, or preferential reaction of generated reactive species with the latter. In how far the presence of SPIONs in the endolysosomal system differentially affects endosomal/lysosomal function of individual cell lines is hard to estimate, as differences in signal intensities for lysosomal proteins between whole cell lysates and lysosome-enriched samples could be due to several factors including enrichment of lysosomal subpopulations, influences of sample complexity on peptide ionization, or differences in loaded sample amounts to the analytical column.

For the proteomic analysis of lysosome-enriched fractions, we identified fairly similar numbers of proteins for all cell lines. Surprisingly, we did not observe general trends concerning the number of identified (lysosomal) proteins (Figs. 2, *C* and *D* and S3, *E* and *F*), implying that no striking fundamental differences exist in the lysosomal composition between the cell lines. When we investigated protein identification across all cell lines, we detected a highly reproducible core proteome of 2173 proteins, of which 308 have been previously reported to be lysosomal (supplemental Table S2). The conserved nature of the latter makes these proteins possible key components for lysosomal function. As the comparison of samples based on protein identification fails to detect subtle changes in expression levels, we performed intensity-based quantification of our data. To allow for comparison of samples originating from human and mouse cells, which result in different peptides for a given protein, we utilized the iBAQ value, which is an estimate of total abundance (49). We only considered proteins that are flagged as lysosomal in public databases (supplemental Table S2) and were reproducibly identified (≥3 replicates each for ≥2 individual cell lines).

In order to compensate for variability between samples, we performed a replicate-wise normalization on a set of V-ATPase subunits that were detected in all analyses of lysosome-enriched fractions with ≥10 peptides. Even though it was shown that the cytosolic V1 part of the V-ATPase is able to dissociate from the V0 part integrated in the lysosomal membrane (49), we did not detect major differences in variability and/or iBAQ values between the subunits of both parts of the complex. This implies that the V-ATPase complex was recovered in its intact state on SPIONs-enriched lysosomes, which is in accordance to previous findings from our group (18). These data also revealed, that the associated subunits *ATP6AP1/2* are lower in abundance and more variable across cell lines with the exception of HeLa cells, in which they were present at similar abundances (supplemental Fig. S5*A*). Interestingly, this observation was only partially replicated on a whole cell lysate level (supplemental Fig. S6*A*). While both subunits also presented with a high variability in this dataset, they were more abundant than others. This implies that, in comparison with other subunits of the complex, a lower percentage of the total pool of these proteins is located at lysosomes.

For the *BLOC*-one-related complex (*BORC*), which is involved in microtubule-mediated lysosomal transport (74), we detected most subunits at highly similar levels within a given cell line but a high variability between cell lines. This implies that the stoichiometry of the complex is conserved, while the number of *BORC* complexes associating with lysosomes is highly dynamic. Interestingly, this pattern was not reproduced on a whole cell proteome level, implying that not all proteins related to the BORC complex are located at lysosomes at a given time. This is in line with reports that several of its subunits serve a dual function and can be located either at lysosomes or mitochondria (75). With respect to individual cell lines, these differences could be related to the fact that lysosomal distribution mechanisms can be heterogeneous, resulting in subpopulations of lysosomes with different moving patterns (16), and that different types of complexes can be responsible for lysosomal positioning, which are partially redundant (6). Furthermore, we detected an ∼8-fold higher abundance of *ARL8B* relative to *ARL8A* and the other *BORC* components. This is probably related to the involvement of ARL8B in other processes, such as, *e.g.*, the recruitment of the *HOPS* tethering complex (76), increasing the amount of lysosome-localized *ARL8B* beyond that associated to *BORC*.

For *mTORC1*, we identified distinct patterns across the lysosome-enriched fractions of the analyzed cell lines, while expression levels were more conserved on a whole cell level. Intriguingly, the members of the Ragulator complex, which consists of *LAMTOR1-5* and is responsible for the recruitment of *mTORC1* to the lysosomal surface (8), were present at lysosomes at higher levels than, *e.g.*, the RAG GTPases or the *mTOR* kinase itself. This may be due to Ragulator’s other roles, as, *e.g.*, lysosomal positioning, due to its interaction with *BORC* (77, 78). Along this line, we observed a similar lysosomal abundance pattern for *BORC* subunits (supplemental Fig. S5*C*) and *mTORC1* members (Fig. 4*B*) in the different cell lines. For individual members of Ragulator, it was quite surprising to us to find lysosome-localized *LAMTOR5* at highly variable levels (∼100-fold dynamic range), clearly exceeding the dynamic range observed for the other subunits. It was shown that loss of *LAMTOR2* induces degradation of the other *LAMTOR* proteins (79). While these data imply that correct assembly of Ragulator is crucial for the stability of its members, our data indicate that this may not be the case for *LAMTOR5*. This would render *LAMTOR*5 dispensable for the stability of other members of the Ragulator complex, implying a possible function as rate-limiting subunit for the full activation of Ragulator (assuming that all *LAMTORs* are needed for Ragulator activity).

For complexes consisting of lysosomal transmembrane transporters and their accessory subunits, we found roughly equal stoichiometries in lysosome-enriched fractions, confirming their direct relationship. This was, for example, the case for *CLCN7* and its β-subunit *OSTM1*, which facilitate Cl^-^ import into lysosomes (80). For these proteins, we found in all cell lines except NIH3T3 a difference in abundance of <2-fold. The same applied for *ATRAID* and *SLC37A3*, which form a complex releasing nitrogen-containing bisphosphonate from lysosomes (81). For *MFSD1* and its accessory subunit *GLMP* (82), however, we detected a heterogeneous pattern with similar stoichiometry in HeLa and SH-SY5Y cells, while the other cell lines showed a consistently higher abundance of *GLMP* (up to 22-fold in case of HuH-7 cells), indicating probable other roles for *GLMP* in certain cell types. Intriguingly, also on a whole cell proteome level we observed highly conserved levels of *MFSD1* in all cell lines and high variability of *GLMP* (Figs. 5*A* and S6*B*). Finally, for the complex of the lysosomal cobalamin transporter *ABCD4* and the lysosomal membrane protein *LMBRD1*, which was shown to facilitate the transport of *ABCD4* from the ER to lysosomes (83), we observed a significantly higher abundance of *ABCD4* (5- to 26-fold) throughout all cell lines. This indicates that *LMBRD1* may be important for the transport of *ABCD4*, but not for its stability or function, as a roughly similar abundance would have to be maintained in this case.

A possible reason for the observed heterogeneity among cell lines could be transcriptional control of lysosomal protein expression. This could be either due to the activity of TFEB and its regulation by different upstream kinases (10) or the activity of other factors affecting lysosomal protein expression such as the members of the FoxO family of transcription factors or the transcriptional repressors ZKSCAN3 or BRD4 (84, 85, 86, 87). Another possibility could be differences in localization. Based on the correlation of protein levels in lysosome-enriched samples and whole cell lysates (supplemental Fig. S7), this seems only to be the case for a subpopulation of proteins, as signal intensities of most proteins correlate in a similar way between both sample types.

Overall, the iBAQ data from both lysosome-enriched samples and whole cell lysates should provide a valuable resource to the community. They present an estimate of abundance for lysosomal proteins in different cell types and allow to estimate the lysosome-localized population for individual proteins. This could allow, for example, to investigate lysosomal heterogeneity in response to certain stimuli or upon alteration of transcription factor activity. Also, for the conceptualization of experiments, this should allow to choose appropriate model systems, as overexpression of the same protein may result in one cell line in a 2-fold higher abundance, while it will result in a 200-fold increase in another. This applies especially to certain metabolic pathways in the individual cell lines, as, for example, we observed extremely high relative levels of most lysosomal enzymes in HuH-7 cells, while concentrations of channels, transporters, and exchangers were rather average.

For the correlation of iBAQ values and Western blot analysis, we observed similar trends in abundance for the majority of protein/sample combinations: 80% (whole cell lysates) and 85% (lysosome-enriched samples) of individual trends matched. The remaining cases, in which we failed to observe a correlation, could be due to several reasons. For the iBAQ analyses, all peptides across the protein sequence are considered. If a certain region of a given protein cannot be covered for a distinct cell line, *e.g.*, due to proteolytic processing, it does not strongly influence the iBAQ value if high intensity peptides from another region are present or if peptides from this region are not utilized at all. If, however, the epitope that is recognized by a specific antibody is affected, the ability of the antibody to detect the respective protein will be heavily impaired. The same is true for posttranslational modifications, such as glycosylation, which are known to be present at many lysosomal luminal protein domains (88, 89), or alterations strongly affecting the size of the protein, therefore resulting in a different migration pattern in SDS-PAGE. This lack of correlation in certain instances is another example for the well-known problem of lack of correlation between MS- and Western blot–based protein quantification (90, 91). It can be appreciated, however, that the Western blot analyses conducted in this study confirm that distinct lysosomal proteins are present at varying levels in individual cell lines, both in whole cell lysates and lysosome-enriched fractions.

To facilitate the identification of previously unknown lysosomal proteins from our datasets, we applied a novel strategy based on the inclusion of differentially SILAC-labeled background cells (which did not receive SPIONs) and bimodal distribution analysis of the resulting samples. While we were able to significantly deplete unspecifically binding proteins from our datasets and to define a population that was specific for the presence of SPIONs (Fig. 6), we were not able to fully remove proteins from certain cellular compartments (Fig. 7*A*). When combining the data of all six cell lines, almost 4500 proteins were categorized as potentially novel lysosomal, representing >20% of known human/mouse proteins.

As it is extremely unlikely that all these proteins play a role with respect to the lysosomal compartment, a large fraction of them has to be considered as nonlysosomal proteins that were reproducibly coenriched. This is most likely due to the transient interaction of lysosomes with other organelles/structures, as it is well established that they form direct contact sites, for example, with the ER, mitochondria, or the plasma membrane (6). For such coenriched interacting organelles, only a certain number of proteins will present true interactors facilitating lysosomal contact (and therefore enrichment), while others are unspecifically coenriched through presence at, or secondary interaction with, the respective structure. In our current analysis, we neglected such proteins for the identification of novel lysosomal proteins due to practical reasons, as the number of candidates would otherwise have been too high to deal with. This practice could, however, also result in removal of true-positive interactors, which are therefore not part of our list of top candidates. These proteins, which also were specifically enriched based on our *p*-value cutoff, can be identified in the individual bimodal distribution analyses of the cell lines (supplemental Table S9). A further possibility is that such identifications could be related to proteins representing substrates degraded in the lysosomal lumen at the point of SPIONs enrichment.

The detection of secondary interactors or partially digested lysosomal substrates should occur with a higher variability than the identification of functionally important lysosomal proteins. We therefore argued that the reproducibility of enrichment, and therefore frequency of identification across the individual datasets, should be lower for such proteins. Consequently, true lysosomal localization should correlate with reproducibility of identification, which was the case for putative lysosomal proteins (Fig. 7, *B* and *C*), and also for such that were previously identified to be present in lysosome-enriched fractions (supplemental Table S12). Based on this assumption, we grouped proteins that were not included in public databases to be localized at lysosomes (supplemental Table S2) and generated a list of potentially novel lysosomal proteins. For reasons of feasibility, we further focused only on such detected in ≥5 cell lines for the selection of targets for follow-up studies (Table 1). It is highly likely that also proteins that we identified in fewer cell lines present high confidence targets, as we observed a certain heterogeneity of expression for several putative/known lysosomal proteins (Fig. 3). Therefore, this list presents a valuable resource for future studies investigating novel lysosomal proteins, as it facilitates the assessment of the likelihood of their localization based on both the SILAC ratio relative to control cells and the frequency of detection across the individual datasets.

Finally, we validated lysosomal localization for six potentially novel lysosomal proteins by overexpression of tagged proteins. For two of them, *SLC31A1* and *NDFIP2*, localization to the lysosomal compartment had been demonstrated previously (61, 70, 71, 72). For SLC31A1, the analysis of overexpressing HEK293 cells showed its exclusive localization at the plasma membrane and endosomes (69), while colocalization with lysosomes was detected in HeLa *VPS35* knock out cells (70). The authors of the latter study concluded that lysosomal localization of *SLC31A1* was presumably related to its missorting, resulting in lysosomal degradation rather than a lysosome-related biological function. On the other hand, subcellular analysis of rat liver organelles demonstrated localization of *SLC31A1* at both lysosomes and the plasma membrane (61), and we observed a clear colocalization with the lysosomal marker protein *LAMP2* in both the transient and stable transfected cell lines (Figs. 8 and S11). Therefore, further studies will be needed to delineate if *SLC31A1* localizes at least partially to the lysosomal membrane under physiological conditions. For the other four validated proteins, no lysosomal localization or lysosome-related functions have been demonstrated yet, making them bona fide lysosomal candidate proteins.

## Data Availability

The mass spectrometry proteomics data have been deposited to the ProteomeXchange Consortium *via* the PRIDE (92) partner repository and are publicly available (http://proteomecentral.proteomexchange.org/) with the dataset identifier PXD020600. MS/MS spectrum identifications from MaxQuant searches were deposited at MS-Viewer (93) and are publicly available (https://msviewer.ucsf.edu/) with the search keys c0vxdeqfnr (combined human datasets from HEK293, HeLa, HuH-7, and SH-SY5Y cells) and qwtwxcxfbm (combined mouse datasets from MEF and NIH3T3 cells).

## Supplemental data

This article contains supplemental data (20, 24, 39, 59, 60, 61, 62, 94).

## Conflict of interest

The authors declare no competing interests.
