# Supplementary material for: Multi–Cell Line Analysis of Lysosomal Proteomes Reveals Unique Features and Novel Lysosomal Proteins
Source: Mol Cell Proteomics. 2023 Feb 14;22(3):100509. doi: 10.1016/j.mcpro.2023.100509 (PMC10025164; doi:10.1016/j.mcpro.2023.100509)
Supplement: Supplementary figures [file mmc15.pdf]

Multi Cell Line Analysis of Lysosomal Proteomes Reveals Unique Features and Novel Lysosomal Proteins

Fatema Akter[1, 2, #], Sara Bonini[1, #], Srigayatri Ponnaiyan[1, #], Bianca Kögler-Mohrbacher[3], Florian Bleibaum[4], Markus Damme[4], Bernhard Y. Renard[3, †], Dominic Winter[1, *]

[1]Institute for Biochemistry and Molecular Biology, Medical Faculty, University of Bonn, Bonn 53115, Germany
[2]Department of Pharmacology, Faculty of Veterinary Science, Bangladesh Agricultural University, Mymensingh 2202, Bangladesh
[3]Bioinformatics Unit (MF1), Robert Koch Institute, Berlin 13353, Germany
[4]Institute for Biochemistry, University of Kiel, Kiel 24118, Germany

#Equal contribution.

*Corresponding author.
Email: dominic.winter@uni-bonn.de (Winter D)

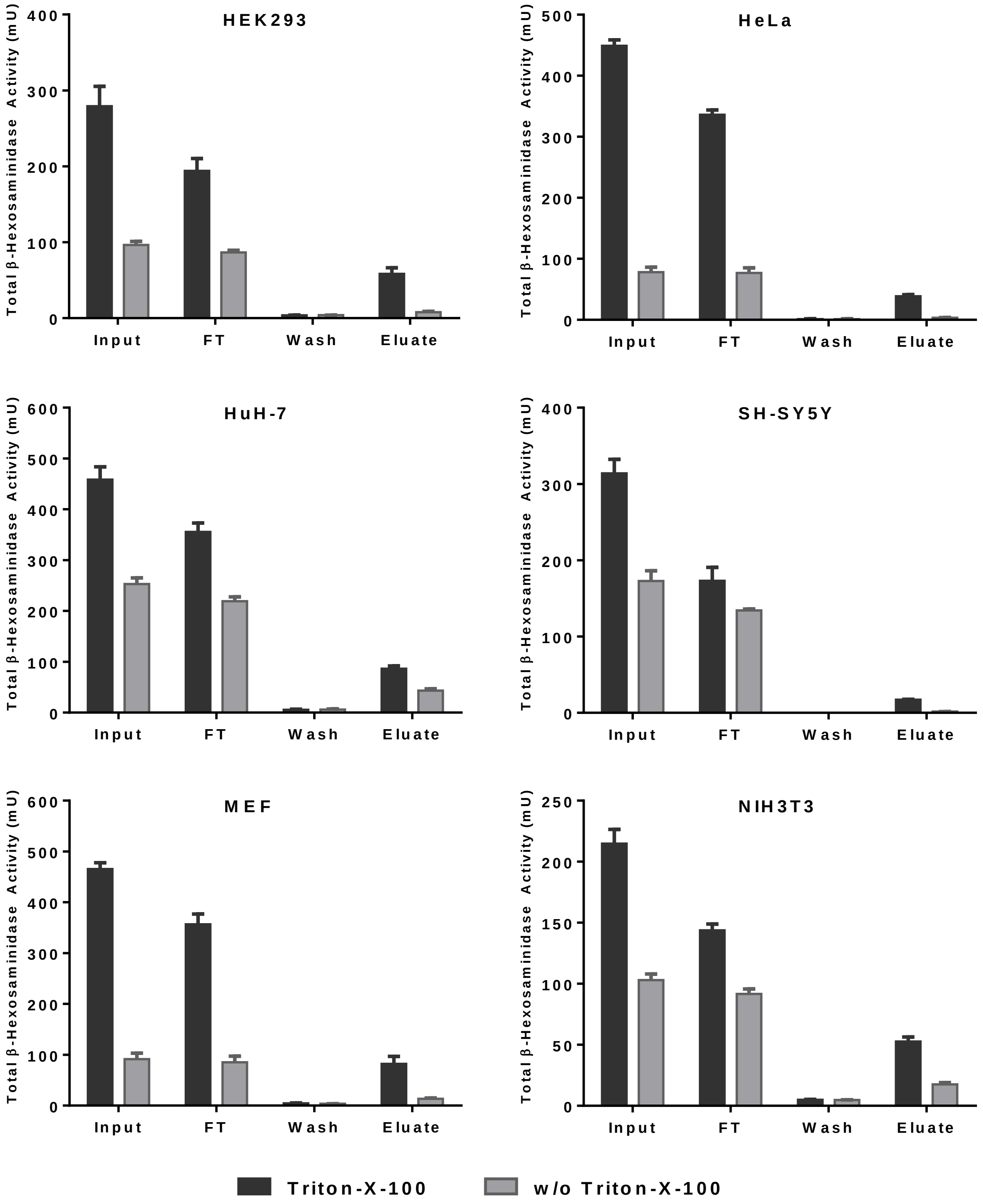

Figure S1 Lysosomal recovery and stability for different cell types

Shown are total β-hexosaminidase activities determined by enzyme activity assays for individual fractions of SPIONs-based lysosome isolation from a 1:1 mixture of differentially SILAC labelled control and SPIONs receiving cells. Comparison of samples with and without Triton-X-100 allows for estimation of percentage of intact lysosomes. FT: flow through, mU: milli units; SILAC: stable isotope labelling by amino acids in cell culture; SPIONs: superparamagnetic iron oxide nanoparticles. Shown are mean values ± SD, n = 4.

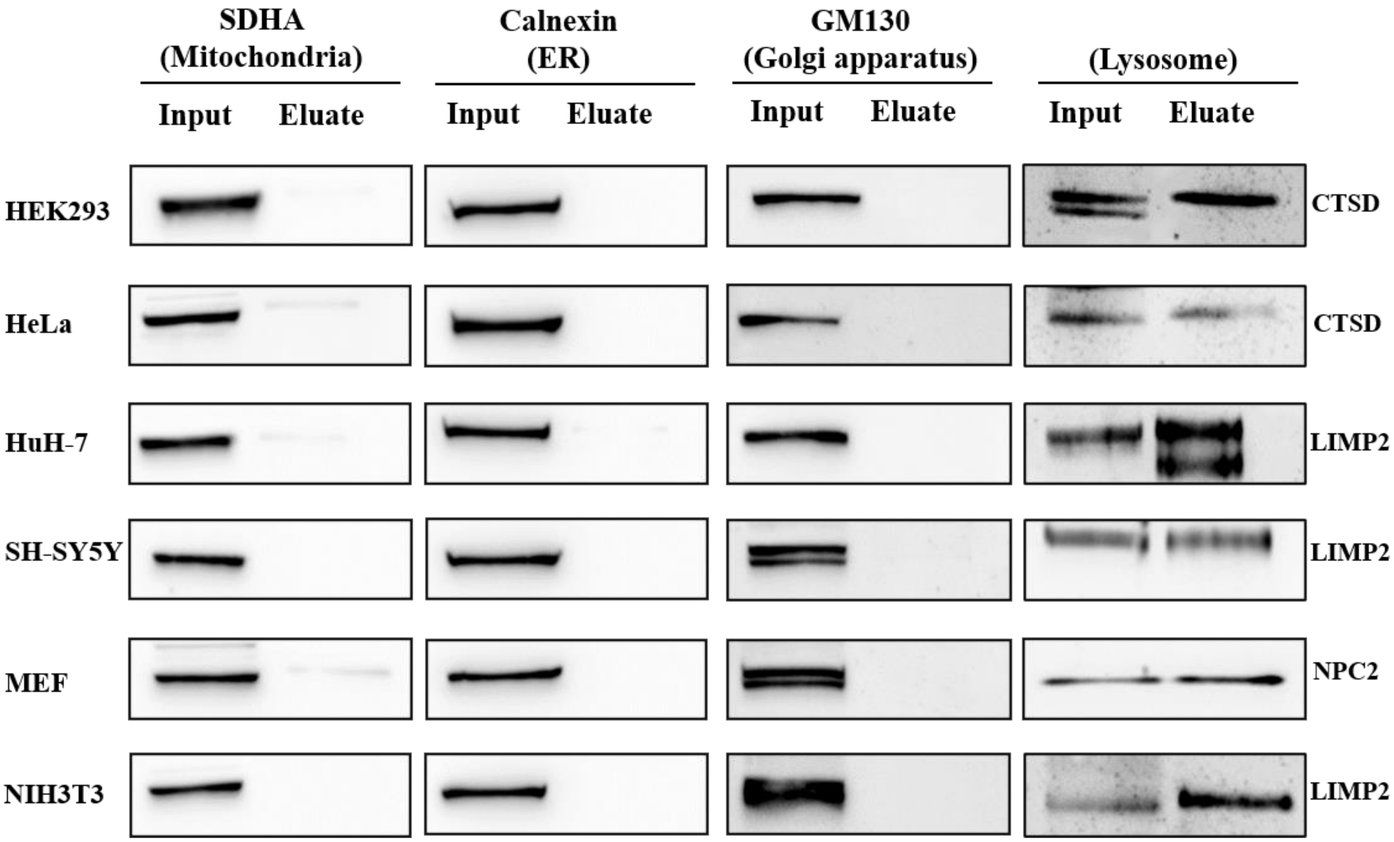

Figure S2 Lysosome enrichment with SPIONs results in lysosome-enriched fractions with low amounts of other contaminating organelles

Shown are western blot analyses from input and eluate fractions of lysosome enrichment experiments from six different cell lines using SPIONs. Markers for mitochondria (SDHA-70kDa), ER (Calnexin-100kDa), Golgi apparatus (GM130-130kDa) and lysosomes (CTSD-36kDa, LIMP2-85kDa, NPC2-15kDa) were analyzed by western blot using the respective antibodies (Table S12). SPIONs, superparamagnetic iron oxide nanoparticles; ER, endoplasmic reticulum; SDHA, Succinate Dehydrogenase Complex Flavoprotein Subunit A; GM130, Golgi matrix protein GM130; CTSD, Cathepsin D; LIMP2, Lysosome membrane protein 2; NPC2, Niemann Pick C type 2.

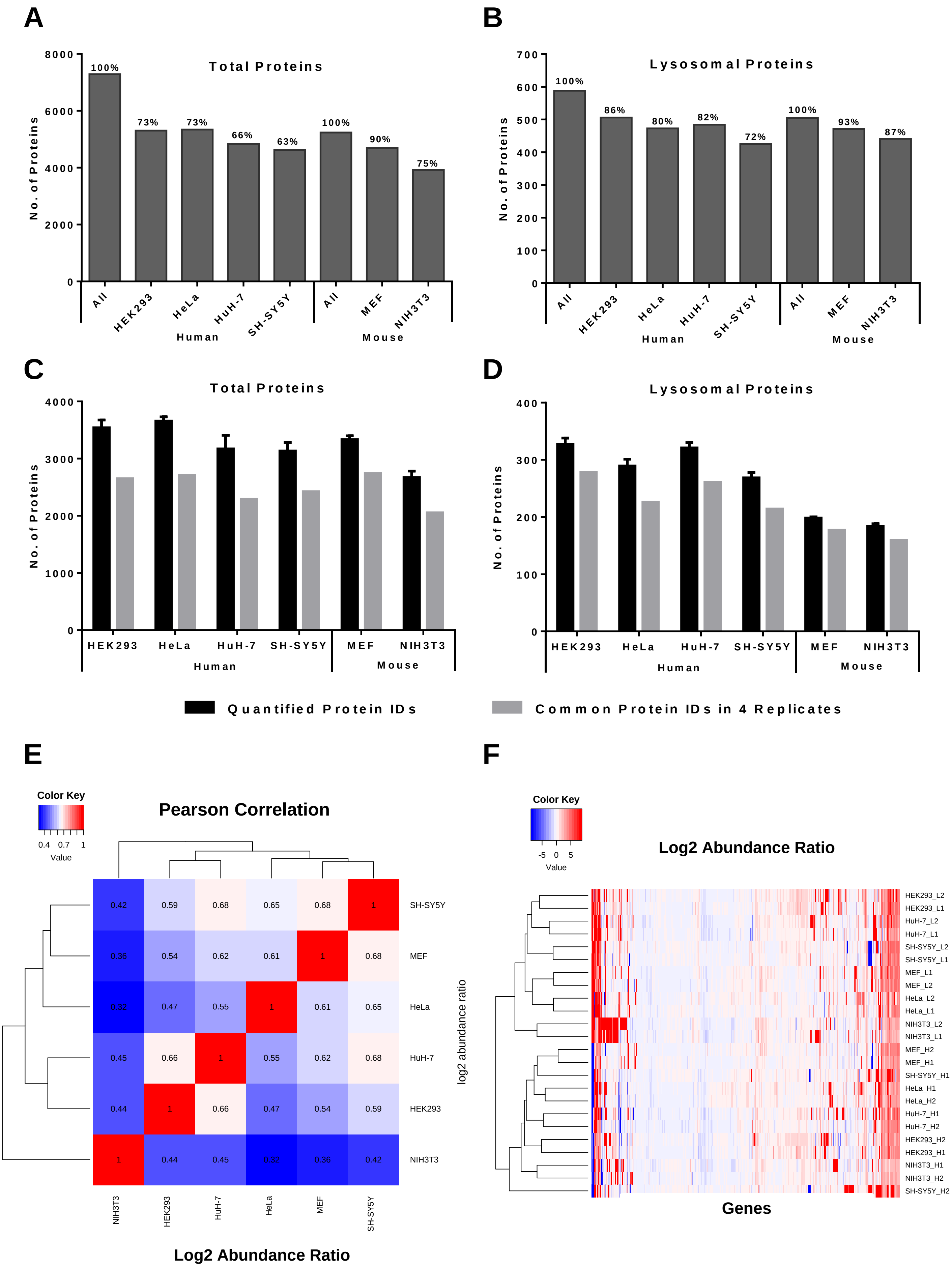

A
Total Proteins
No. of Proteins
100%
73%
73%
66%
63%
100%
90%
75%
All
HEK293
HeLa
HuH-7
SH-SY5Y
All
MEF
NIH3T3
Human
Mouse
B
Lysosomal Proteins
No. of Proteins
100%
86%
80%
82%
72%
100%
93%
87%
C
Total Proteins
D
Lysosomal Proteins
Quantified Protein IDs
Common Protein IDs in 4 Replicates
E
Color Key
Value
Pearson Correlation
Log2 Abundance Ratio
log2 abundance ratio
SH-SY5Y
MEF
HeLa
HuH-7
HEK293
NIH3T3
F
Log2 Abundance Ratio
Genes
HEK293_L2
HEK293_L1
HuH-7_L2
HuH-7_L1
SH-SY5Y_L2
SH-SY5Y_L1
MEF_L1
MEF_L2
HeLa_L2
HeLa_L1
NIH3T3_L2
NIH3T3_L1
MEF_H2
MEF_H1
SH-SY5Y_H1
HeLa_H1
HeLa_H2
HuH-7_H1
HuH-7_H2
HEK293_H2
HEK293_H1
NIH3T3_H1
NIH3T3_H2
SH-SY5Y_H2

Figure S3 Analysis of lysosome-enriched fractions by mass spectrometry based proteomics

A. Total protein numbers and identification rates relative to the sum of proteins identified in the whole study for the datasets of the individual cell lines. **B.** Total numbers and identification rates relative to the sum of known lysosomal proteins identified in the whole study of known lysosomal proteins for the individual cell lines. **C.** Numbers of quantified proteins and such detected in all four biological replicates for the datasets of the individual cell lines. **D.** Numbers of quantified proteins and such detected in all four biological replicates for known lysosomal proteins in the individual cell lines. **E.** Pearson correlation values of log2 abundance ratios (SPIONs/control) for binary comparisons of individual cell lines based on their mean values (n=4). **F.** Heat map and hierarchical clustering of log2 abundance ratios (SPIONs/control). (A-D) Shown are mean values ± SD, n = 4; SPIONs: superparamagnetic iron oxide nanoparticles.

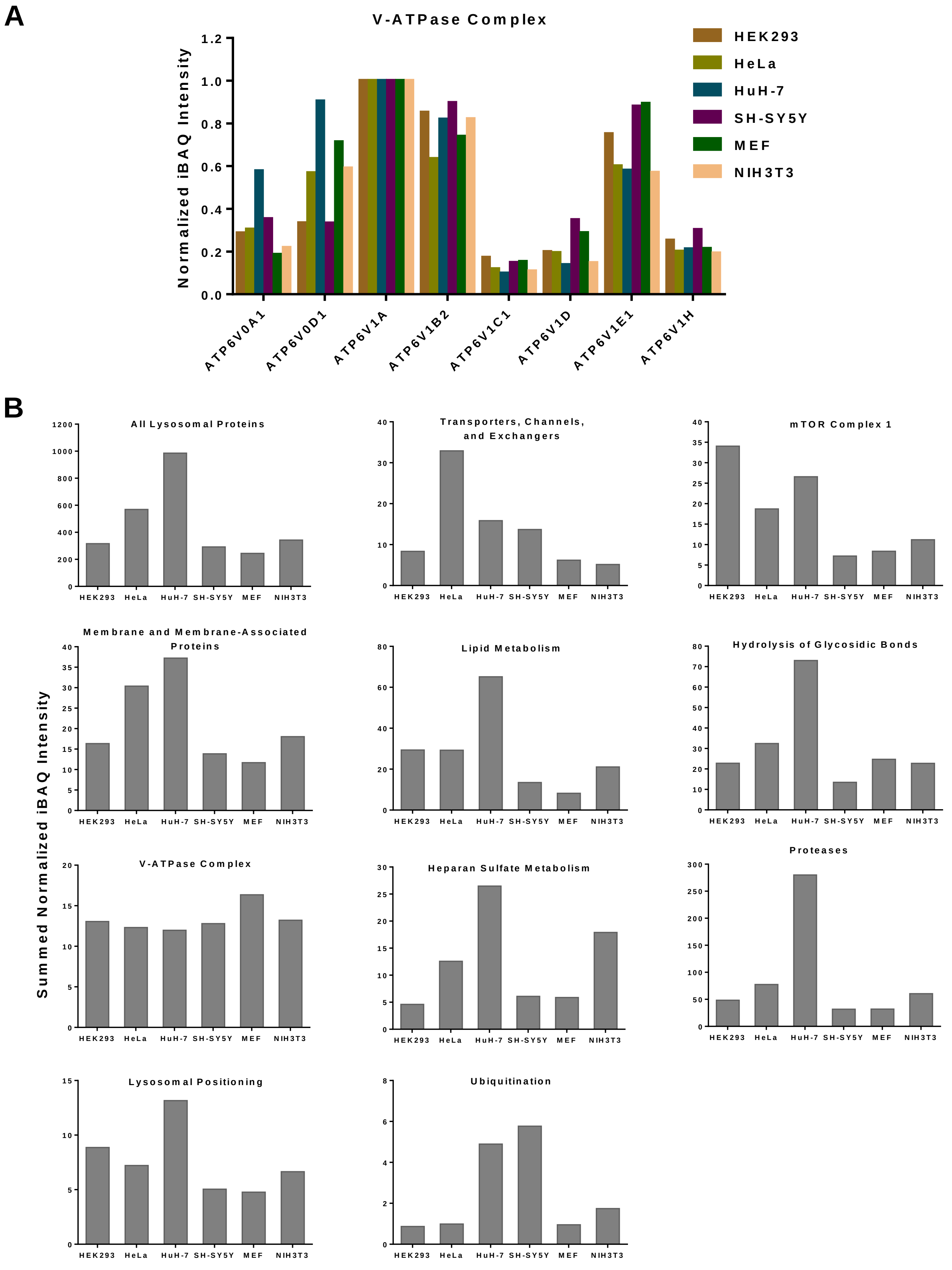

A
V-ATPase Complex
Normalized iBAQ Intensity
0.0
0.2
0.4
0.6
0.8
1.0
1.2
HEK293
HeLa
HuH-7
SH-SY5Y
MEF
NIH3T3
ATP6V0A1
ATP6V0D1
ATP6V1A
ATP6V1B2
ATP6V1C1
ATP6V1D
ATP6V1E1
ATP6V1H
B
Summed Normalized iBAQ Intensity
All Lysosomal Proteins
Transporters, Channels, and Exchangers
mTOR Complex 1
Membrane and Membrane-Associated Proteins
Lipid Metabolism
Hydrolysis of Glycosidic Bonds
V-ATPase Complex
Heparan Sulfate Metabolism
Proteases
Lysosomal Positioning
Ubiquitination
HEK293
HeLa
HuH-7
SH-SY5Y
MEF
NIH3T3

Figure S4 Analysis of protein abundance levels by intensity Based Absolute Quantification (iBAQ).

A. Subunits of the V-ATPase complex which were detected in all cell lines with ≥ 10 unique peptides. Shown are the median iBAQ intensities (n = 4) normalized to the expression of ATP6V1A. **B.** Summed normalized iBAQ intensities for distinct categories of known lysosomal proteins. iBAQ values of individual proteins were normalized to the median intensity of the eight V-ATPase subunits displayed in (A) in a replicate-wise manner. Subsequently, the mean of 4 biological replicates was calculated for each individual protein and the summed values were calculated. Only proteins present in ≥3 replicates of ≥2 cell lines were included.

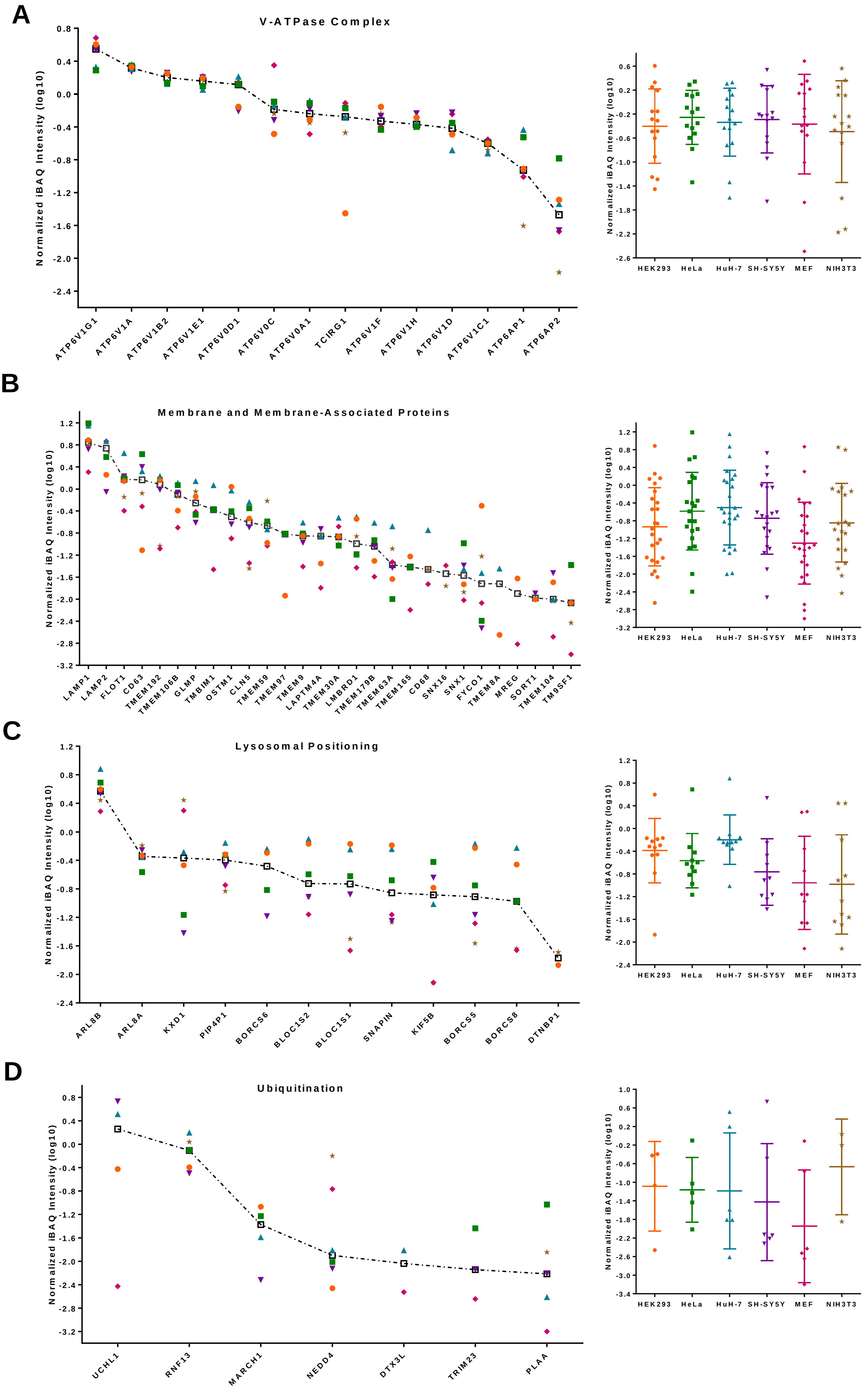
A
V-ATPase Complex
Normalized iBAQ Intensity (log10)
0.8
0.4
0.0
-0.4
-0.8
-1.2
-1.6
-2.0
-2.4
ATP6V1G1
ATP6V1A
ATP6V1B2
ATP6V1E1
ATP6V0D1
ATP6V0C
ATP6V0A1
TCIRG1
ATP6V1F
ATP6V1H
ATP6V1D
ATP6V1C1
ATP6AP1
ATP6AP2
Normalized iBAQ Intensity (log10)
0.6
0.2
-0.2
-0.6
-1.0
-1.4
-1.8
-2.2
-2.6
HEK293
HeLa
HuH-7
SH-SY5Y
MEF
NIH3T3
B
Membrane and Membrane-Associated Proteins
Normalized iBAQ Intensity (log10)
1.2
0.8
0.4
0.0
-0.4
-0.8
-1.2
-1.6
-2.0
-2.4
-2.8
-3.2
LAMP1
LAMP2
FLOT1
CD63
TMEM192
TMEM106B
GLMP
TMBIM1
OSTM1
CLN5
TMEM59
TMEM97
TMEM9
LAPTM4A
TMEM30A
LMBRD1
TMEM179B
TMEM63A
TMEM165
CD68
SNX16
SNX1
FYCO1
TMEM8A
MREG
SORT1
TMEM104
TM9SF1
Normalized iBAQ Intensity (log10)
1.2
0.8
0.4
0.0
-0.4
-0.8
-1.2
-1.6
-2.0
-2.4
-2.8
-3.2
HEK293
HeLa
HuH-7
SH-SY5Y
MEF
NIH3T3
C
Lysosomal Positioning
Normalized iBAQ Intensity (log10)
1.2
0.8
0.4
0.0
-0.4
-0.8
-1.2
-1.6
-2.0
-2.4
ARL8B
ARL8A
KXD1
PIP4P1
BORCS6
BLOC1S2
BLOC1S1
SNAPIN
KIF5B
BORCS5
BORCS8
DTNBP1
Normalized iBAQ Intensity (log10)
1.2
0.8
0.4
0.0
-0.4
-0.8
-1.2
-1.6
-2.0
-2.4
HEK293
HeLa
HuH-7
SH-SY5Y
MEF
NIH3T3
D
Ubiquitination
Normalized iBAQ Intensity (log10)
0.8
0.4
0.0
-0.4
-0.8
-1.2
-1.6
-2.0
-2.4
-2.8
-3.2
UCHL1
RNF13
MARCH1
NEDD4
DTX3L
TRIM23
PLAA
Normalized iBAQ Intensity (log10)
1.0
0.6
0.2
-0.2
-0.6
-1.0
-1.4
-1.8
-2.2
-2.6
-3.0
-3.4
HEK293
HeLa
HuH-7
SH-SY5Y
MEF
NIH3T3

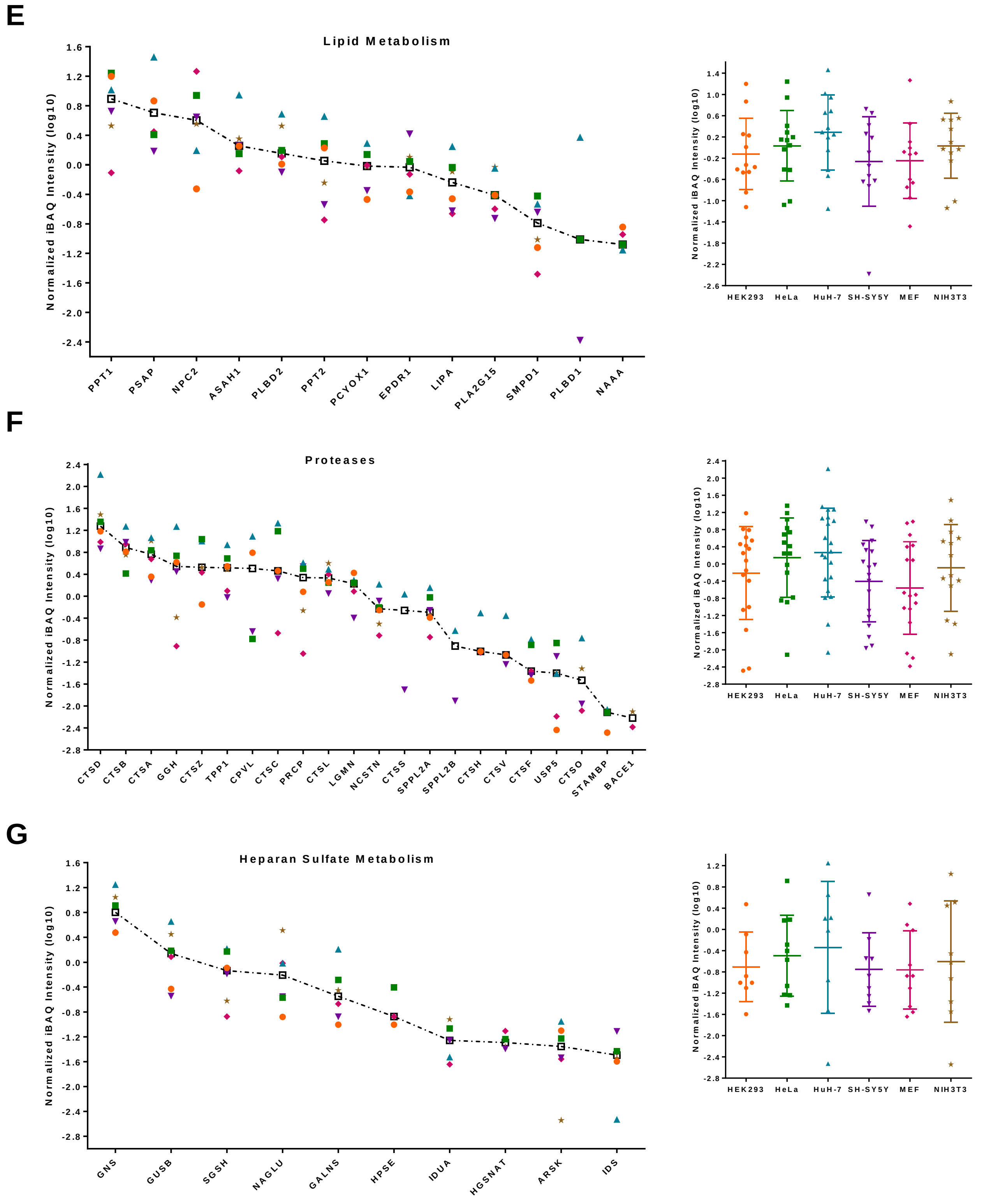

Figure S5 Individual V-ATPase normalized iBAQ values for proteins grouped in categories of similar function.

Shown are median V-ATPase normalized iBAQ values either for individual proteins sorted in descending order based on the median value of expression levels across all cell lines (scatter plots) or for the respective cell line (dotted box plots). **A.** Proteins related to the V-ATPase complex. **B.** Membrane and membrane-associated proteins. **C.** Selected proteins known to play a role in lysosomal positioning, members of the BORC complex. **D.** Lysosome-associated cytosolic ubiquitin ligases. **E.** Proteins known to play a role in lysosomal lipid metabolism. **F.** Lysosomal proteases. **G.** Proteins involved in the degradation of heparan sulfate.

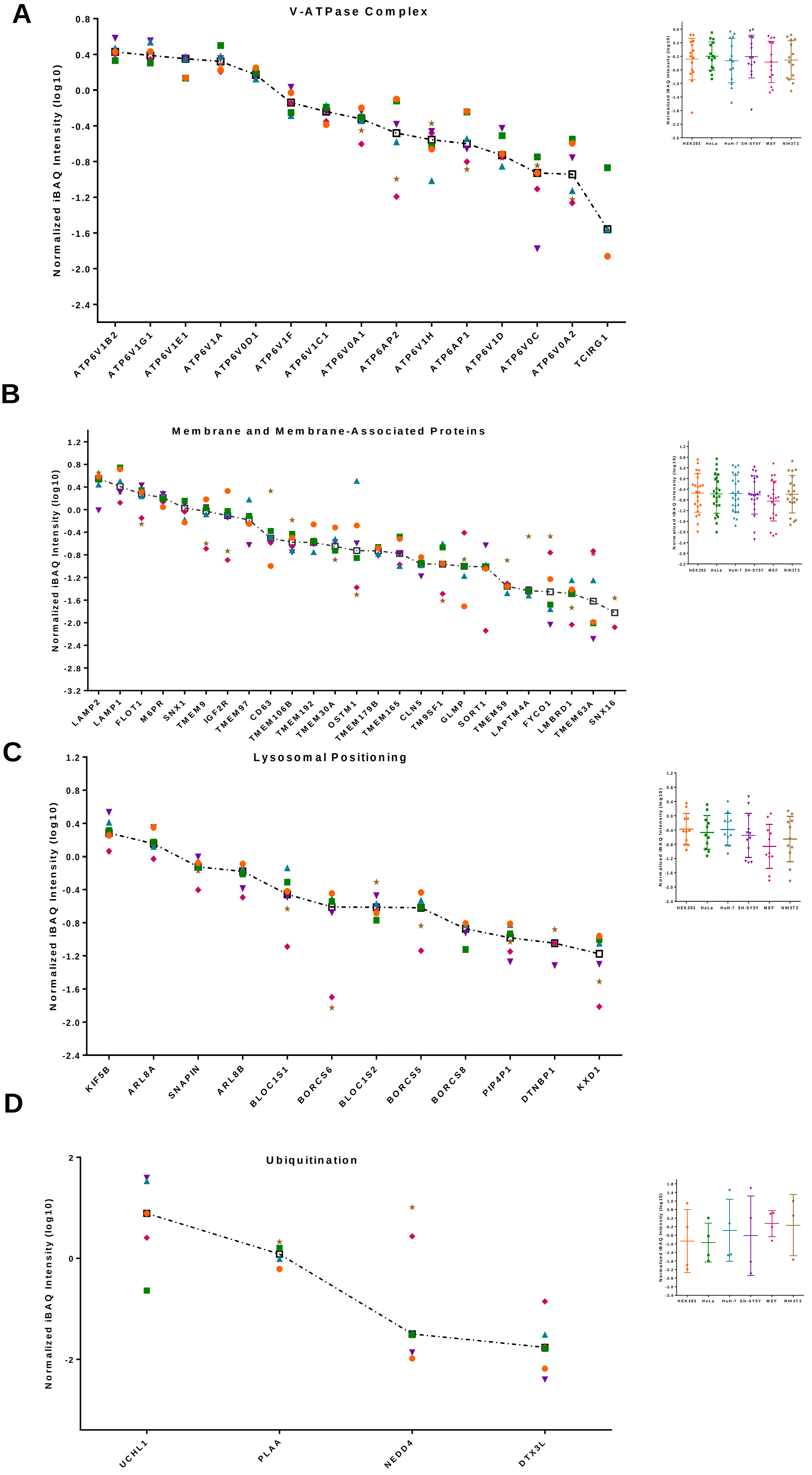
A
V-ATPase Complex
Normalized iBAQ Intensity (log10)
0.8
0.4
0.0
-0.4
-0.8
-1.2
-1.6
-2.0
-2.4
ATP6V1B2
ATP6V1G1
ATP6V1E1
ATP6V1A
ATP6V0D1
ATP6V1F
ATP6V1C1
ATP6V0A1
ATP6AP2
ATP6V1H
ATP6AP1
ATP6V1D
ATP6V0C
ATP6V0A2
TCIRG1
HEK293
HeLa
HuH-7
SH-SY5Y
MEF
NIH3T3
B
Membrane and Membrane-Associated Proteins
Normalized iBAQ Intensity (log10)
1.2
0.8
0.4
0.0
-0.4
-0.8
-1.2
-1.6
-2.0
-2.4
-2.8
-3.2
LAMP2
LAMP1
FLOT1
M6PR
SNX1
TMEM9
IGF2R
TMEM97
CD63
TMEM106B
TMEM192
TMEM30A
OSTM1
TMEM179B
TMEM165
CLN5
TM9SF1
GLMP
SORT1
TMEM59
LAPTM4A
FYCO1
LMBRD1
TMEM63A
SNX16
C
Lysosomal Positioning
Normalized iBAQ Intensity (log10)
1.2
0.8
0.4
0.0
-0.4
-0.8
-1.2
-1.6
-2.0
-2.4
KIF5B
ARL8A
SNAPIN
ARL8B
BLOC1S1
BORCS6
BLOC1S2
BORCS5
BORCS8
PIP4P1
DTNBP1
KXD1
D
Ubiquitination
Normalized iBAQ Intensity (log10)
2
0
-2
UCHL1
PLAA
NEDD4
DTX3L

E

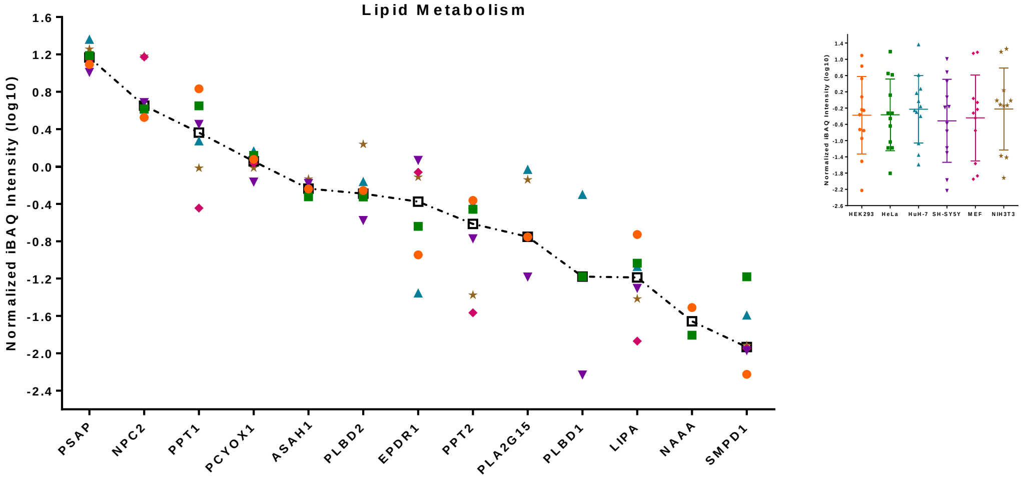

F

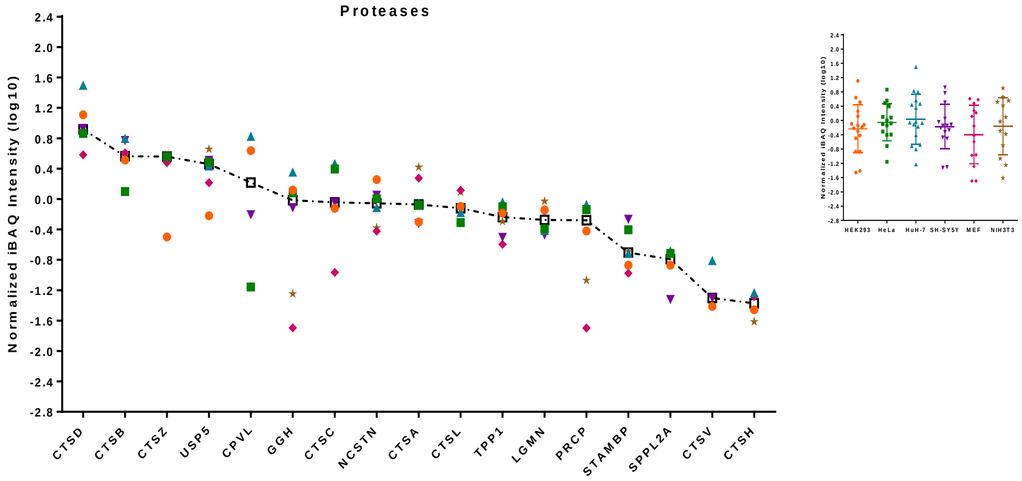

G

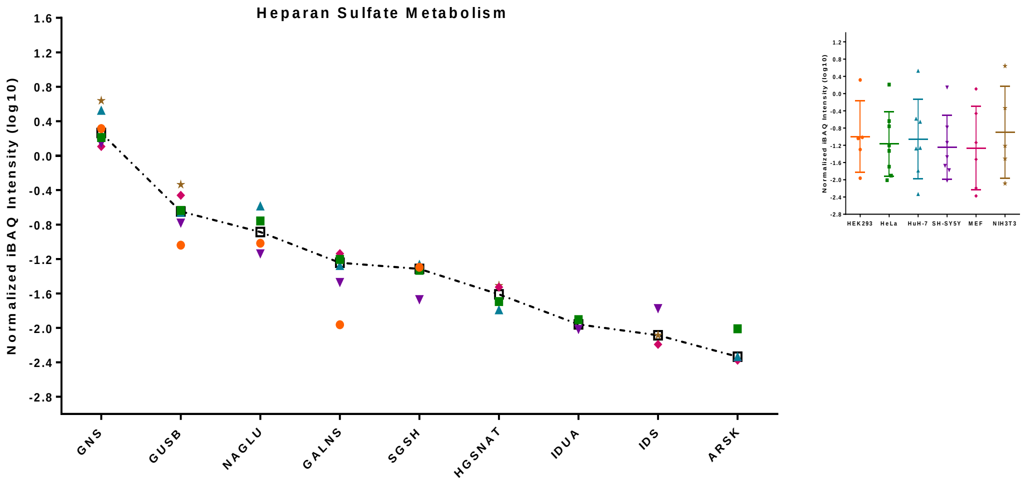

Figure S6 V-ATPase normalized iBAQ values determined from DIA analyses of whole cell lysates for proteins grouped in categories of similar function.

Shown are median V-ATPase normalized iBAQ values either for individual proteins sorted in descending order based on the median value of expression levels across all cell lines (scatter plots) or for the respective cell line (dotted box plots). **A.** Proteins related to the V-ATPase complex. **B.** Membrane and membrane-associated proteins. **C.** Selected proteins known to play a role in lysosomal positioning, members of the BORC complex. **D.** Lysosome-associated cytosolic ubiquitin ligases. **E.** Proteins known to play a role in lysosomal lipid metabolism. **F.** Lysosomal proteases. **G.** Proteins involved in the degradation of heparan sulfate.

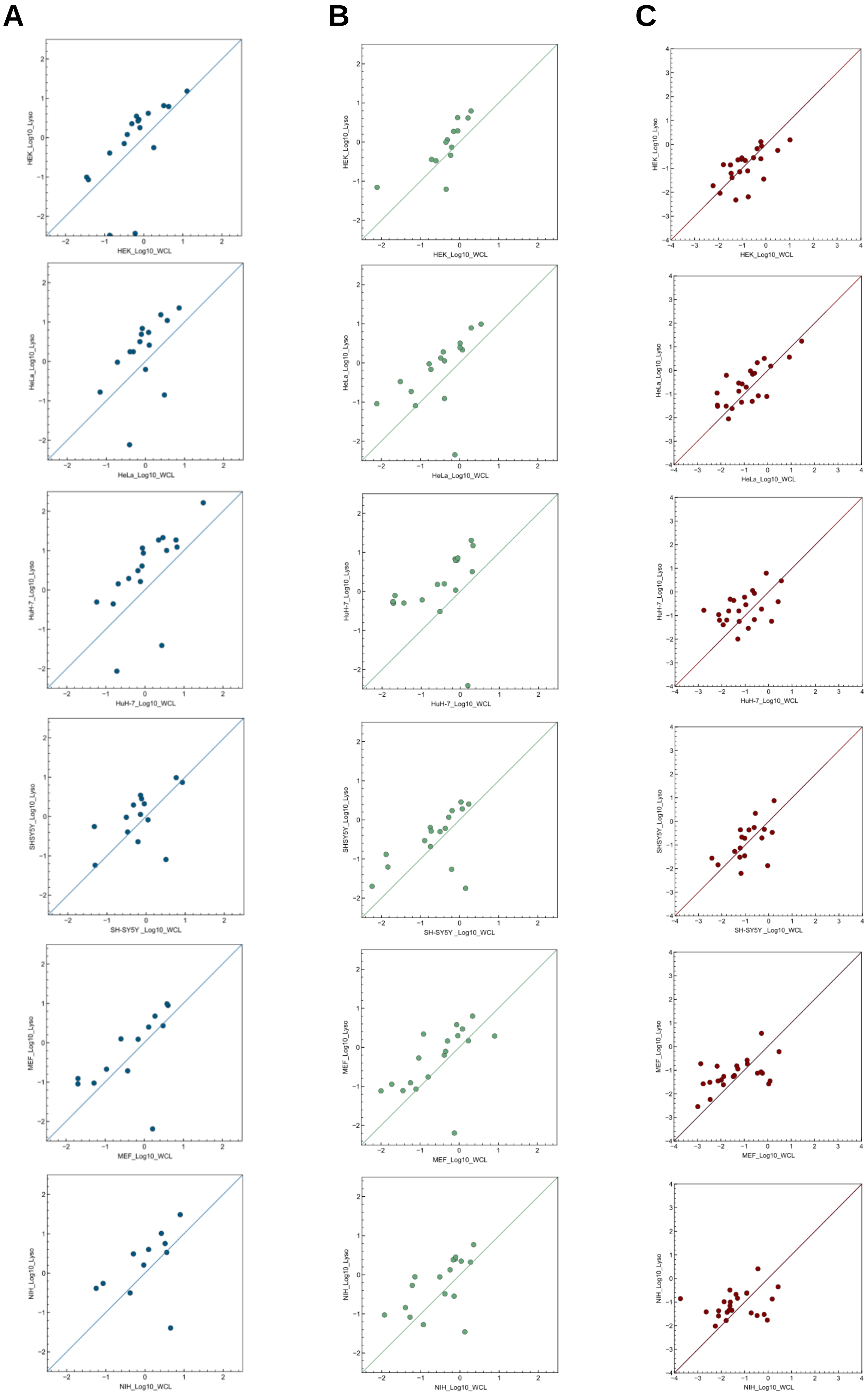
A
B
C
HEK_Log10_Lyso
HEK_Log10_WCL
HeLa_Log10_Lyso
HeLa_Log10_WCL
HuH-7_Log10_Lyso
HuH-7_Log10_WCL
SHSY5Y_Log10_Lyso
SH-SY5Y _Log10_WCL
MEF_Log10_Lyso
MEF_Log10_WCL
NIH_Log10_Lyso
NIH_Log10_WCL

Figure S7 Correlation of iBAQ values between whole cell lysates and lysosome-enriched fractions

Log10 transformed V-ATPase normalized iBAQ values determined for whole cell lysates (x axis) and lysosome-enriched fractions (y-axis). Data are shown for three subcategories of lysosomal proteins: **A** Proteases **B** Hydrolysis of glycosidic bonds and **C** Transporters, channel and exchangers. The figure was generated with Instant Clue v. 0.10.10.20210316.

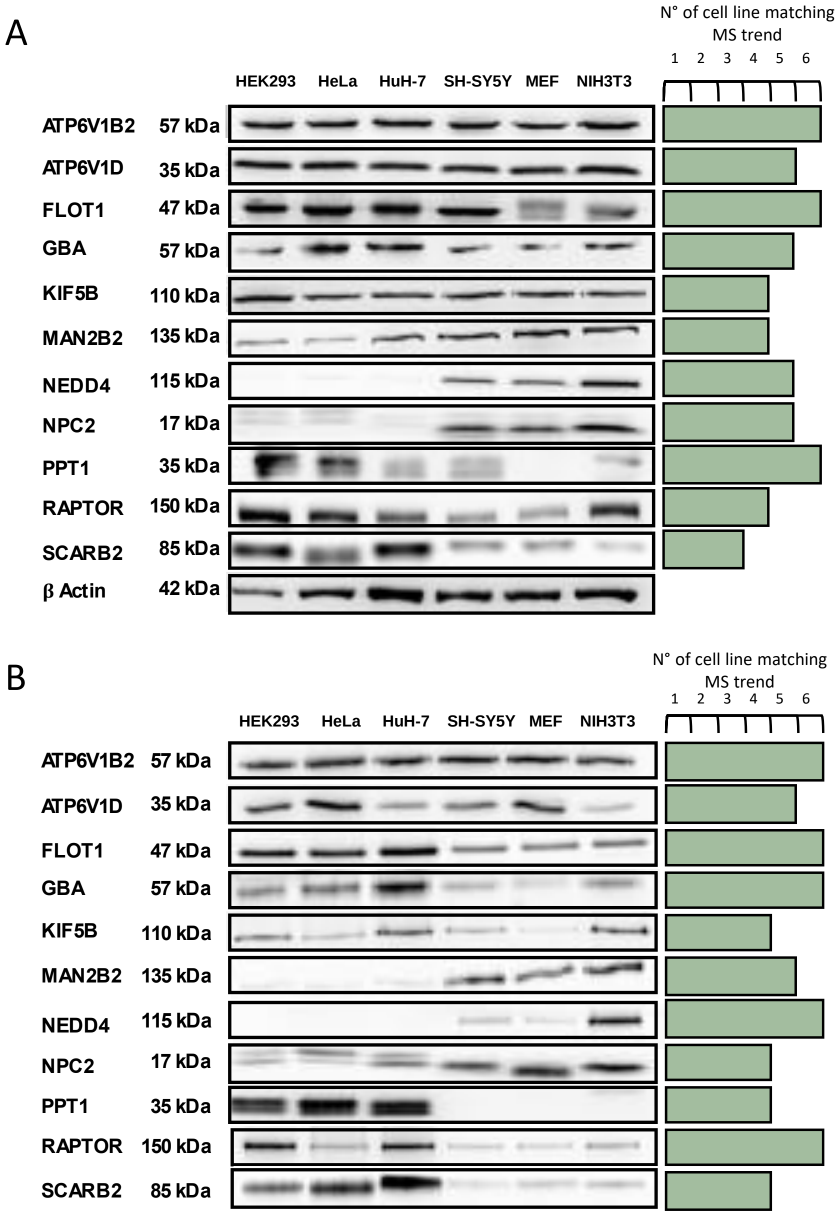

Figure S8 Western blot analysis of cell line-specific differences in the expression levels of lysosomal proteins

Western blot analyses of lysosomal protein abundance in whole cell lysates and lysosome-enriched fractions from six different cell lines. **A.** Analysis of whole cell lysate fractions, equal amounts of protein were loaded for each lane. **B.** Analysis of lysosome-enriched fractions, loaded protein amounts were normalized based on the signal intensity for ATP6V1B2, similar to the normalization of mass spectrometry data. Trends observed in all western blot analyses were compared to both V-ATPase-normalized mass spectrometry data sets of whole cell lysates and lysosome-enriched fractions (see Figure 4, 5 and Figure S5, 6). Next to each panel of blots, the number of bands fitting to the mass iBAQ values is indicated. Densitometric quantification values, iBAQ values, and numbers of unique peptides identified for individual proteins can be found in Table S14.

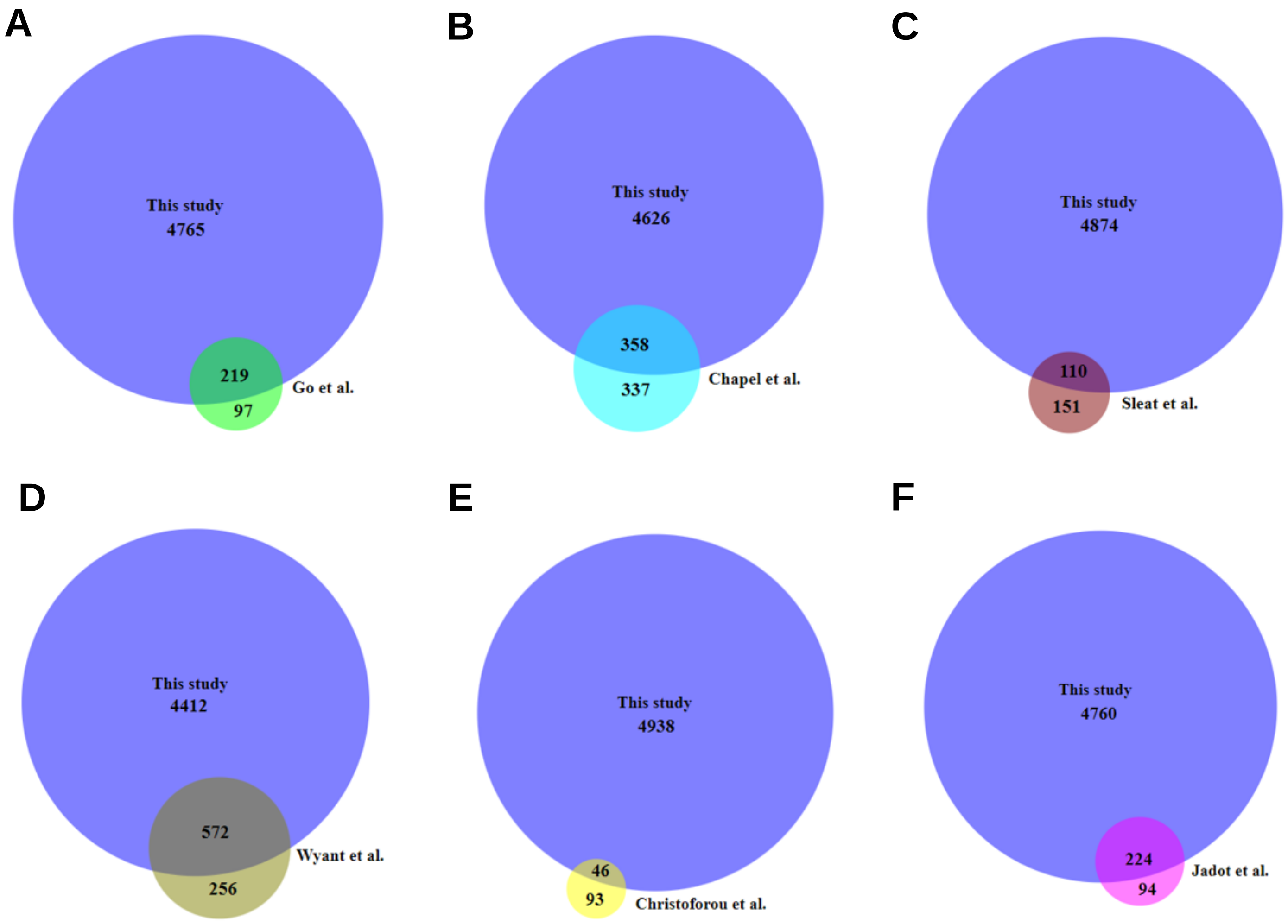

Figure S9 Potential novel lysosomal proteins identified by bimodal distribution analysis show a high overlap with previously published potential lysosomal proteins

Potentially novel lysosomal proteins identified in this study were compared to published datasets originating from the proteomic analysis of lysosome-enriched fractions or the analysis of cell-wide protein distribution patterns (see Table S10). For this study, all proteins were considered for the comparison which were significantly enriched in at least one cell line based on the bimodal distribution analysis. **A.** Putative lysosomal proteins identified by Go et al. using proximity biotinylation in HEK293 cells [2]. **B.** Potential novel lysosomal proteins identified by Chapel et al. in a lysosomal membrane fraction from rat liver [3]. **C.** Putative lysosomal proteins identified by Sleat et al. in acid phosphatase-deficient mice [4]. **D.** Proteins found to be significantly enriched in immunoprecipitated HEK293 lysosomes by Wyant et al. [5]. **E.** Potential novel lysosomal proteins based on their distribution behaviour in density gradient centrifugation experiments performed by Christoforou et al. in E14TG2a murine ES cells [6]. **F.** Proteins identified to be potentially located at lysosomes by Jadot et al. employing subcellular fractionation of rat liver samples [7]. Venn diagrams were generated using BioVenn [8].

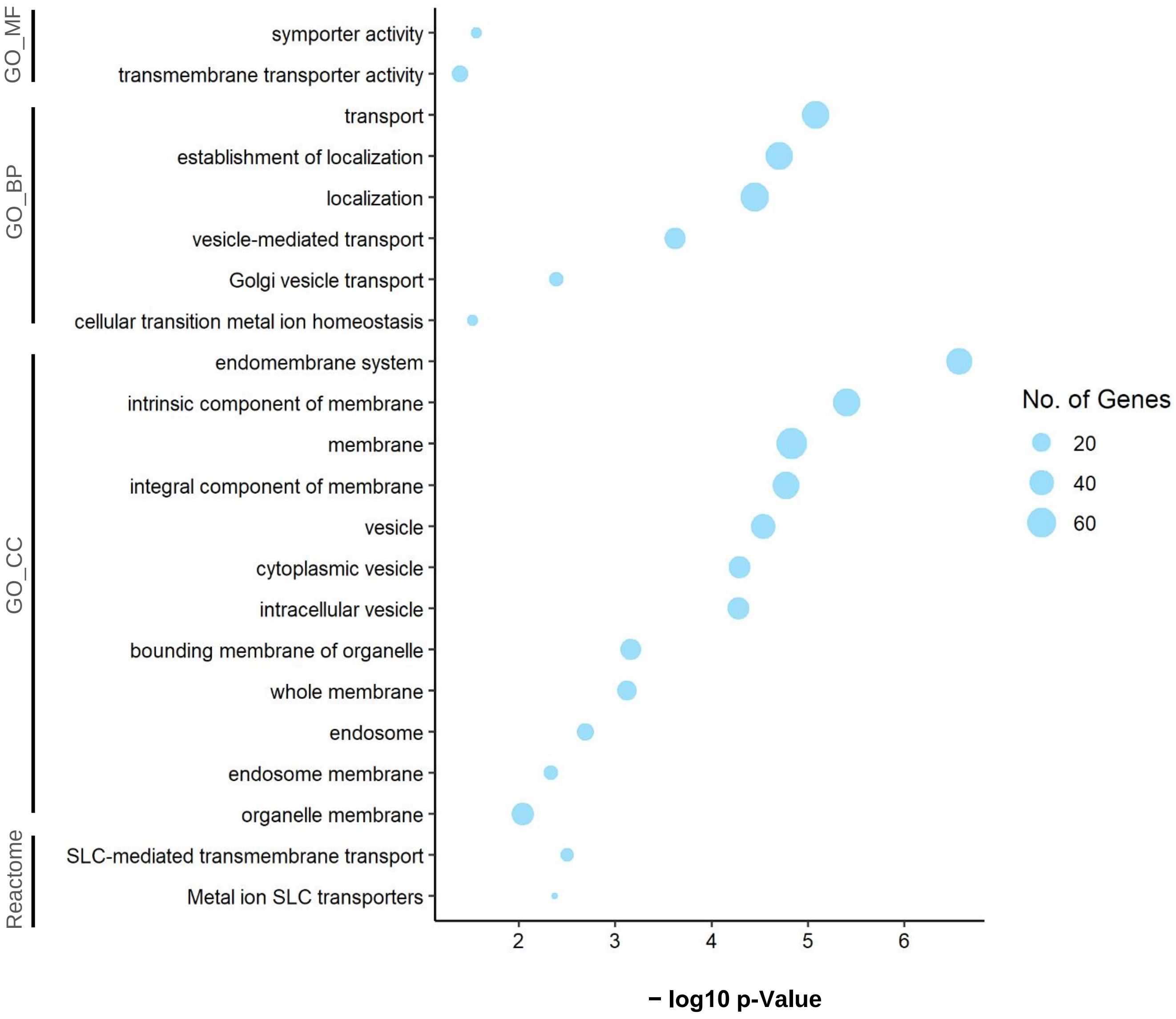

Figure S10 Gene Ontology analysis of high confidence potential novel lysosomal proteins. Proteins which were identified to be significantly overrepresented in SPIONs receiving cells (p-value < 0.05) of ≥ 5 individual cell lines were subjected to GO and reactome pathway analyses. Bubble size correlates with the number of genes assigned to the respective category. GO: gene ontology; MF: molecular function; BP: biological process; CC: cellular component; SPIONs: superparamagnetic iron oxide nanoparticles.

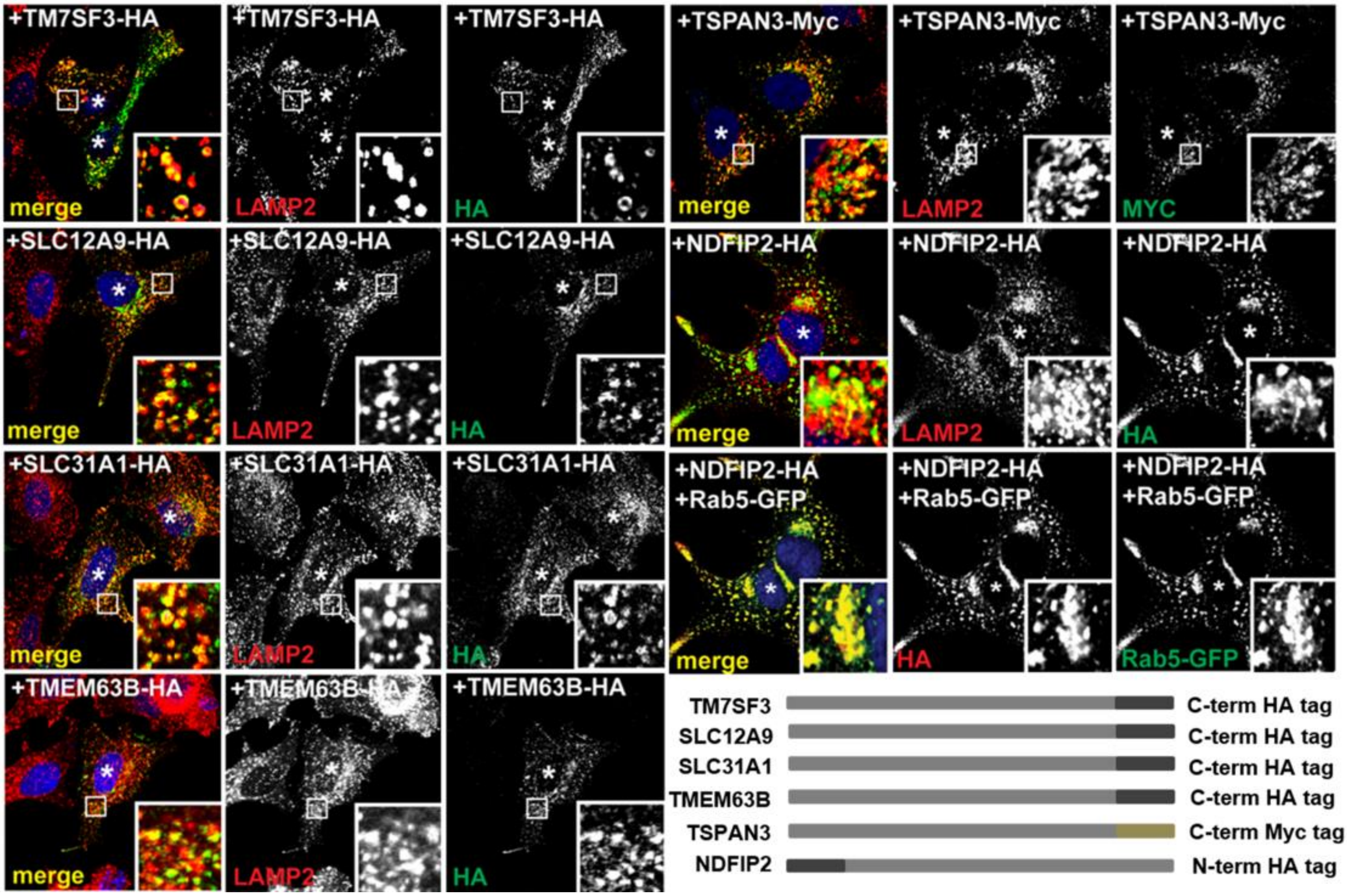

Figure S11 Potential novel lysosomal proteins localize to lysosomes or endosomes

For six proteins detected in the significantly SPIONs enriched fractions of ≥ 5 cell lines, C-/N-terminally tagged constructs were generated. Following transient transfection of HeLa cells, co-localization with lysosomes (LAMP2 staining) or endosomes (Rab5 staining) was investigated by immunocytochemistry. TM7SF3, Transmembrane 7 superfamily member 3; SLC12A9, Solute carrier family 12 member 9; SLC31A1, Solute carrier family 31 member 1; TMEM63B, Transmembrane protein 63B; TSPAN3, Tetraspanin 3; NDFIP2, Nedd4 family interacting protein 2; Rab5, RAS associated protein 5; GFP, Green fluorescent protein; LAMP2, Lysosomal associated membrane protein 2.
